# Maternal obesity alters histone modifications mediated by the interaction between EZH2 and AMPK, impairing neural differentiation in the developing embryonic brain cortex

**DOI:** 10.1016/j.jbc.2025.108173

**Published:** 2025-01-10

**Authors:** Thilina T. Alawathugoda, Muhammad Abid Sheikh, Anil Kumar Challagandla, S. Thameem Dheen, Bright Starling Emerald, Suraiya Anjum Ansari

**Affiliations:** 1 Department of Biochemistry, College of Medicine and Health Sciences, United Arab Emirates University, Al Ain, Abu Dhabi, United Arab Emirates; 2 Department of Anatomy, College of Medicine and Health Sciences, United Arab Emirates University, Al Ain, Abu Dhabi, United Arab Emirates; 3 Department of Anatomy, Yong Loo Lin School of Medicine, National University of Singapore, Singapore; 4 Zayed Center for Health Sciences, United Arab Emirates University, Al Ain, Abu Dhabi, United Arab Emirates; 5 ASPIRE Precision Medicine Research Institute Abu Dhabi (PMRI-AD), United Arab Emirates University, Al Ain, United Arab Emirates

**Keywords:** maternal obesity, embryonic neurogenesis, RNA-seq, ChIP-seq, epigenetics

## Abstract

Neurodevelopmental disorders have complex origins that manifest early during embryonic growth and are associated with intricate gene regulation dynamics. A perturbed metabolic environment such as hyperglycemia or dyslipidemia, particularly due to maternal obesity, poses a threat to the optimal development of the embryonic central nervous system. Accumulating evidence suggests that these metabolic irregularities during pregnancy may alter neurogenesis pathways, thereby predisposing the developing fetus to neurodevelopmental disorders. One primary mechanism through which such disruptions may occur involves changes in histone modifications resulting from fluctuations in the expression of histone-modifying enzymes or the availability of their substrates. Herein, we have used a rat model of maternal obesity induced by a high-fat diet before and during gestation to investigate the cellular and molecular repercussions of maternal obesity on embryonic cortical neurogenesis. Maternal obesity impairs neurogenesis by reducing cell proliferation, increasing neuronal marker expression, and shifting development toward astrogliogenesis. Differentially expressed genes revealed disruptions in key developmental signaling pathways and reduced AKT phosphorylation, particularly at E14.5. These changes were associated with epigenetic alterations, mainly the differential expression and phosphorylation of EZH2 and subsequent changes in global histone modifications. Chromatin immunoprecipitation sequencing revealed reduced H3K27me3 at genes upregulated due to maternal obesity, which could have resulted from reduced expression and increased phosphorylation of EZH2 at Thr311. Interestingly, EZH2 also showed increased O-GlcNAcylation in high-fat diet embryos along with increased association with AMPK-Thr172 in accordance with previous studies showing that Ampk catalyzes EZH2-Thr311p. These results suggest that an epigenetic gene regulatory mechanism mediated by Ampk and Ezh2 interactions resulted in reduced H3K27me3 and derepression of key developmental genes, which could have led to cell fate changes observed in the developing embryo brain cortex due to maternal obesity.

Maternal obesity and metabolic syndrome during pregnancy have emerged as crucial factors influencing both maternal and fetal health outcomes. The maternal metabolic environment can impact the developing fetus, potentially leading to an increased risk of obesity, metabolic syndrome, and other health issues in the offspring later in life (1, 2). Furthermore, recent epidemiological research has brought attention to the connections linking maternal obesity and diabetes to neurodevelopmental and psychiatric disorders in the offspring. These investigations underscore the heightened risk of diverse conditions, encompassing autism spectrum disorder, attention-deficit hyperactivity disorder, anxiety, depression, schizophrenia, and other related pathologies (3, 4, 5). The proposed mechanisms of such connections encompass fetal exposure to factors like inflammation, hormonal imbalances, oxidative stress, and disturbances in epigenetic regulation (1, 6). Animal models of high-fat diet (HFD) fed maternal obesity have shed light on such pathological mechanisms; however, a clear understanding of maternal obesity–related neurodevelopmental and neuropsychiatric disorders of the offspring remains obscure (1, 7). One of the fundamental ways the prenatal environment influences the organism is by inducing epigenetic modifications. These modifications entail enduring and heritable alterations to the genome without any changes to the underlying DNA sequence. Epigenetic modifications encompass processes such as DNA methylation, posttranslational histone modifications (including acetylation, methylation, ubiquitylation, and sumoylation), and variations in miRNA expression. The impact of these epigenetic alterations on gene expression is now recognized to significantly influence an individual's lifelong susceptibility to conditions like obesity, metabolic disorders, and cardiovascular and neurological diseases (8). Thus, exposure to an obesogenic *in utero* environment during embryogenesis can induce epigenetic changes in offspring, potentially heightening their vulnerability to disease.

The frontal cortex, particularly the brain's prefrontal cortex, plays a central and critical role in higher executive functions, for example, planning and complex decision-making, social cognition, emotional regulation, and temporal integration of information (9, 10). Numerous psychiatric disorders and neurological conditions are linked to impairments in cognitive control and/or malfunction of the prefrontal cortex and its related neural circuitry (9). It has been proposed that maternal obesity and the offspring's exposure to an HFD may have adverse effects on cognitive function by impacting the hippocampus and prefrontal cortex (11), thus exposing the developing fetus to the risk of neurodevelopmental disorders. Yet, the mechanisms connecting these factors to the pathophysiological alterations in the developing brain remain incompletely understood. Moreover, given the pivotal roles of epigenetic mechanisms in shaping the fate of stem cells during development and subsequent differentiation, it becomes imperative to investigate the aberrant epigenetic gene regulatory mechanisms associated with nutrient imbalances resulting from conditions like hyperglycemia and/or dyslipidemia.

Therefore, to assess the influence of maternal obesity on embryonic cortical neurodevelopment, we have used a rat model of HFD-fed maternal obesity to discern its effects on embryo brain development at cellular and molecular levels. Maternal obesity was noted to markedly disrupt embryonic neurogenesis, not only altering the ratios of cell proliferation to differentiation but also inducing a transition towards astrogliogenesis. Gene expression analysis revealed disruptions in key developmental signaling pathways. The altered AKT signaling pathway, along with epigenetic alterations, namely, the differential expression and phosphorylation of EZH2 and subsequent changes in global histone modifications, advanced our understanding of the profound impact that maternal obesity can exert on developing embryo brain cortex. Therefore, this study effectively showcases the multifaceted consequences of maternal obesity on embryonic neurogenesis, providing comprehensive insights at both the cellular and molecular levels.

## Results

### Maternal obesity increases lipid accumulation and cell death in the developing embryo brain cortex

**Figure 1:**
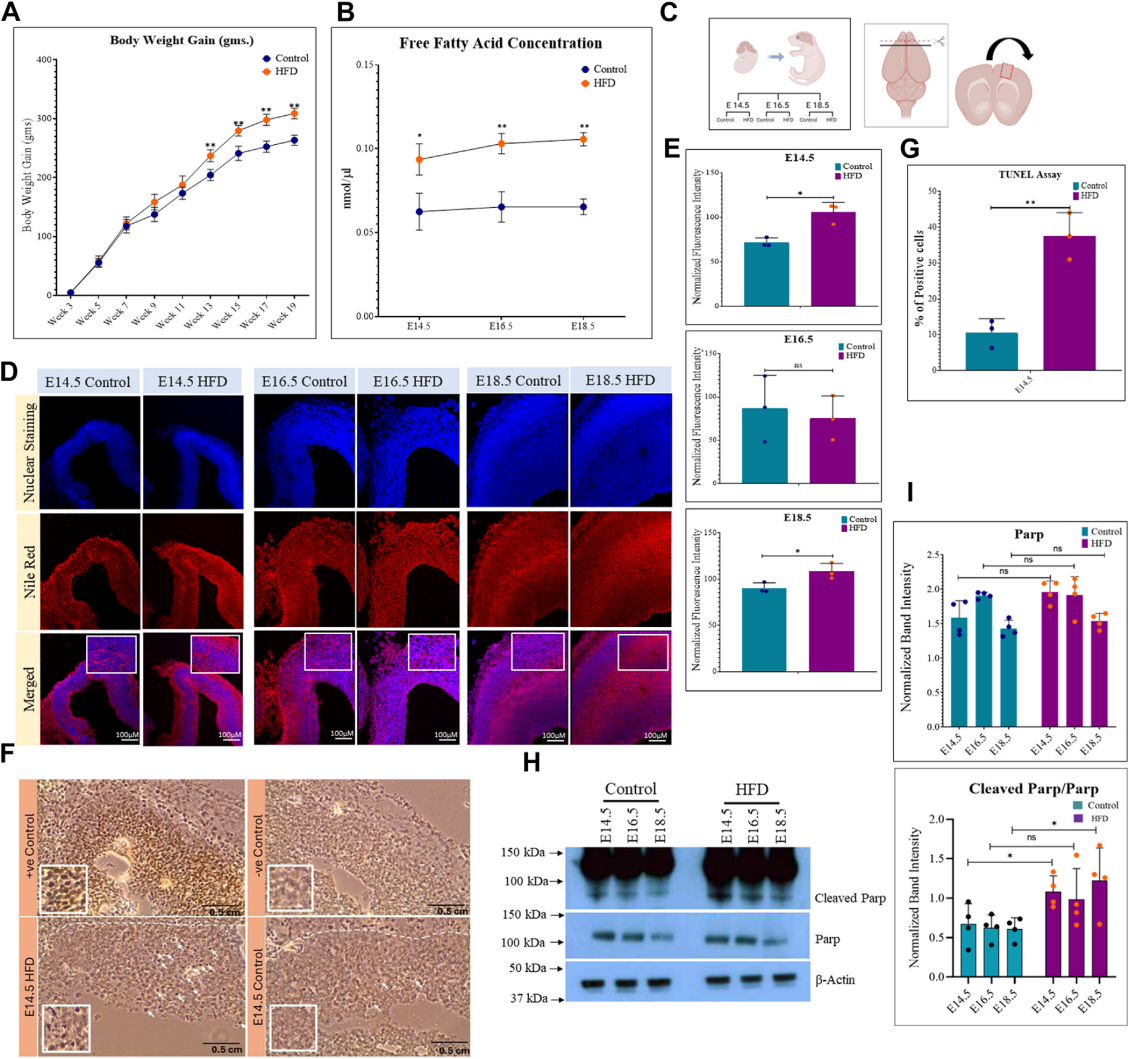
**The impact of maternal high fat diet–induced obesity on embryo brain cortex.***A*, comparative body weight gain (grams) of the control (normal chow) and the HFD groups of female rats prior to and during gestation. *B*, plasma-free fatty acid concentration (nmol/μl) in pregnant rats on control and HFDs at the time of their sacrifice at gestational stages, E14.5, E16.5, and E18.5. *C*, schematic representation of embryo brain isolation and sectioning for immunohistochemistry comparing normal and HFD embryo brains at stages E14.5, E16.5, and E18.5. *D*, measurement of lipid accumulation through Nile Red staining in control and HFD embryo brain cortex at E14.5, E16.5, and E18.5. The scale bar represents 100 μm. *E*, quantitation of Nile Red fluorescence intensity (*panel C*) normalized to 4′,6-diamidino-2-phenylindole. *F*, TUNEL staining of control and HFD embryo brain cortex at E14.5. The scale bar represents 0.5 cm. *G*, TUNEL-positive cells were counted to quantify and analyzed with reference to a positive and negative control in the stained sections. *H*, Western blotting was used for the expression of Parp and cleaved Parp on control and HFD embryo brain cortical tissue lysates at E14.5, E16.5, and E18.5 stages. The expression of β-Actin was used as the loading control. *I*, densitometric quantitation of Western blots from *panel H*:cleaved Parp levels are plotted relative to total Parp. The data (*bars*) are represented as mean ± SD. n = 3. ∗∗*p* < 0.01, ∗*p* < 0.05, n/s, not significant.

Female Wistar rats were randomly selected and divided into two groups, control (fed normal chow and water) and HFD (fed a commercial HFD and water), for approximately 10 weeks postweaning. The HFD-treated group of females became visibly heavier than the control group. Notably, the difference in the body weights between the two groups started to become significant by the 15th week of the feeding cycle (Fig. 1*A*). After which, these animals were mated with males on normal chow. Levels of plasma-free fatty acids (FFAs) in the pregnant animals were assessed at three developmental stages (E14.5, E16.5, and E18.5) before they were sacrificed. The results revealed a significant elevation in FFAs in the obese animals compared to controls (Fig. 1*B*). Embryo brains were removed under a dissection microscope, and lipid accumulation in the frontal lobe sections was examined using Nile red staining at all three stages. We found a significant increase in lipid accumulation in the frontal cortex of embryo brains from obese rats compared to controls at stages E14.5 and E18.5, with no substantial change observed at E16.5 (Fig. 1, *C*–*E*). We then conducted TUNEL assay to assess cell death in the frontal cortex sections. Our findings revealed an increase in TUNEL-positive cells in HFD embryo brains compared to the control at E14.5 (Fig. 1, *F* and *G*). These results were further validated by Western blot analysis for Poly (ADP-ribose) polymerase (PARP) and cleaved PARP, which showed a significant increase in the levels of cleaved PARP at E14.5 and E18.5 stages, whereas changes were not significant at E16.5 (Fig. 1, *H* and *I*). These findings demonstrate that maternal obesity and elevated levels of plasma FFAs contribute to an augmented lipid accumulation in the developing embryo brain cortex, concomitant with an upsurge in cell death.

### Maternal obesity impairs cell differentiation during embryonic cortical neurogenesis

**Figure 2:**
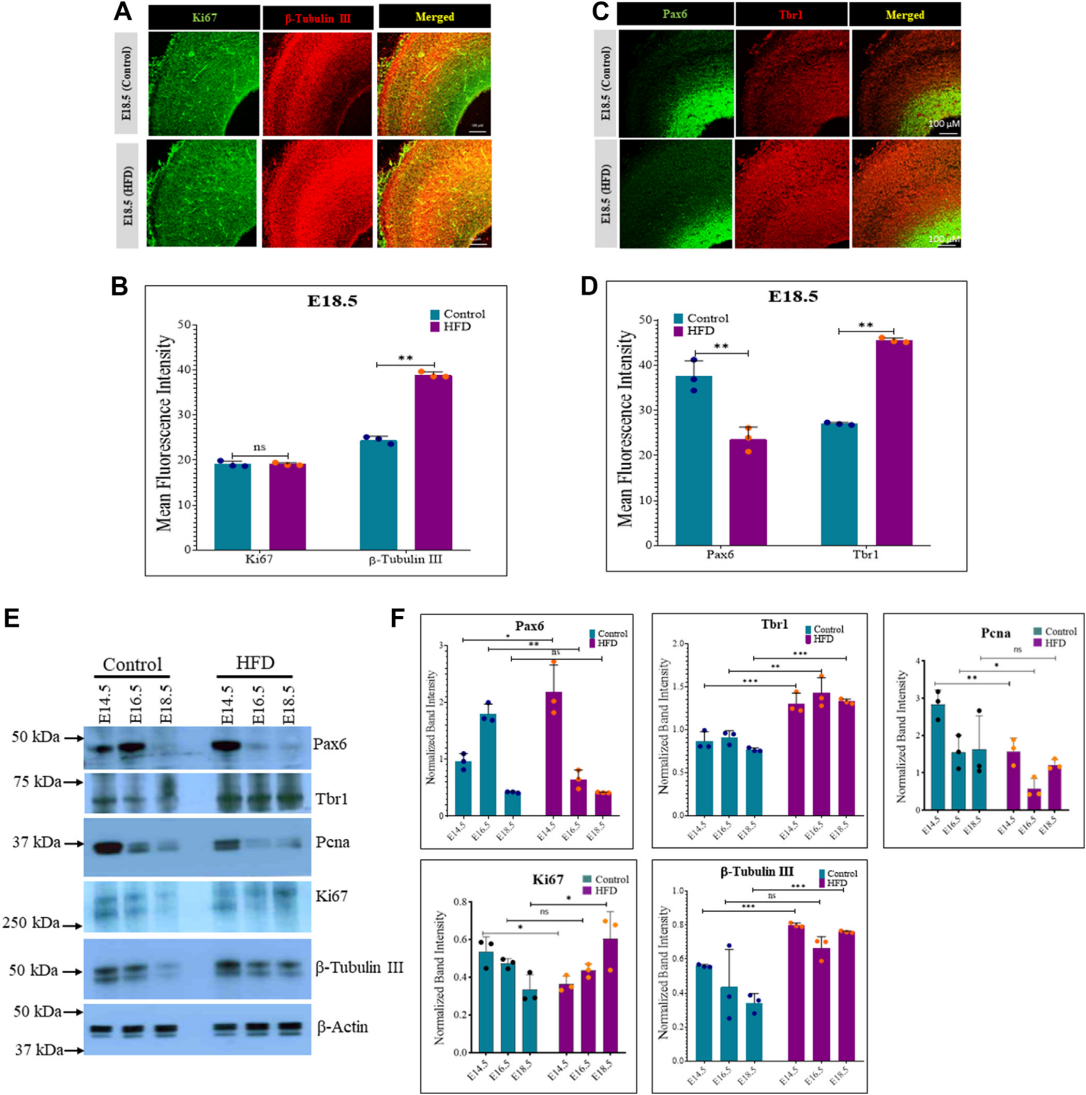
**Maternal obesity causes cell proliferation and differentiation imbalance during embryonic cortical neurogenesis.***A* and *B*, immunostaining in control and HFD embryo brain cortex at E18.5 for cell proliferation marker Ki67 and neuronal marker β-tubulin III (*A*) and neural cell markers, Pax6 and Tbr1 (*B*). The scale bar represents 100 μm. *C* and *D*, fluorescence intensity from *panel A and B* was quantified using ImageJ (Fiji) software and normalized to TO-PRO-3 nuclear staining intensity. *E*, Western blot analysis for the expression of Pax6, Tbr1, Ki67, Pcna, and β-tubulin III on control and HFD embryo brain cortical tissue lysates at E14.5, E16.5, and E18.5 stages. The expression of β-Actin was used as the loading control. *F*, densitometric quantitation of Western blots from *panel E*. n = 3. The data (*bars*) are represented as mean ± SD. ∗∗*p* < 0.01 and ∗*p* < 0.05. n/s, not significant; HFD, high-fat diet.

Next, we investigated the expression of markers related to cell proliferation and differentiation at specific embryonic stages. The frontal lobe sections were immunostained for KI67 and β-TUBULIN III for cell proliferation and neuronal production, respectively. The embryonic brain cortex of obese animals showed an increased β-TUBULIN III expression compared to control, whereas KI67 was unaltered (Fig. 2, *A* and *B*) at E18.5. To correlate these changes with markers and regulators of neural stem cell fate, we analyzed the expression of PAX6 and TBR1, which regulate neural stem cell proliferation and differentiation, respectively, in the developing embryo brain cortex (12). The levels of TBR1 were substantially higher, whereas PAX6 showed a subtle but significant decrease in obese rat embryos at E18.5 than controls (Fig. 2, *C* and *D*). To confirm these changes, Western blotting was performed on the prefrontal cortices of control and obese animals at developmental stages E14.5, E16.5, and E18.5. The expression of TBR1 was higher in obese animals at all three stages, whereas a mixed result was observed for PAX6 expression in Western blots where its expression was higher in HFD at E14.5, decreased at E16.5 and no significant change was observed at E18.5 (Fig. 2, *E* and *F*). The expression of cell proliferation markers KI67 and PCNA was lower in obese animals at E14.5 than controls, with inconsistent changes at the other two stages (Fig. 2, *E* and *F*). Additionally, β-TUBULIN III expression was higher in obese animals at all three stages, though the increase was not statistically significant at E16.5 (Fig. 2, *E* and *F*).

**Figure 3:**
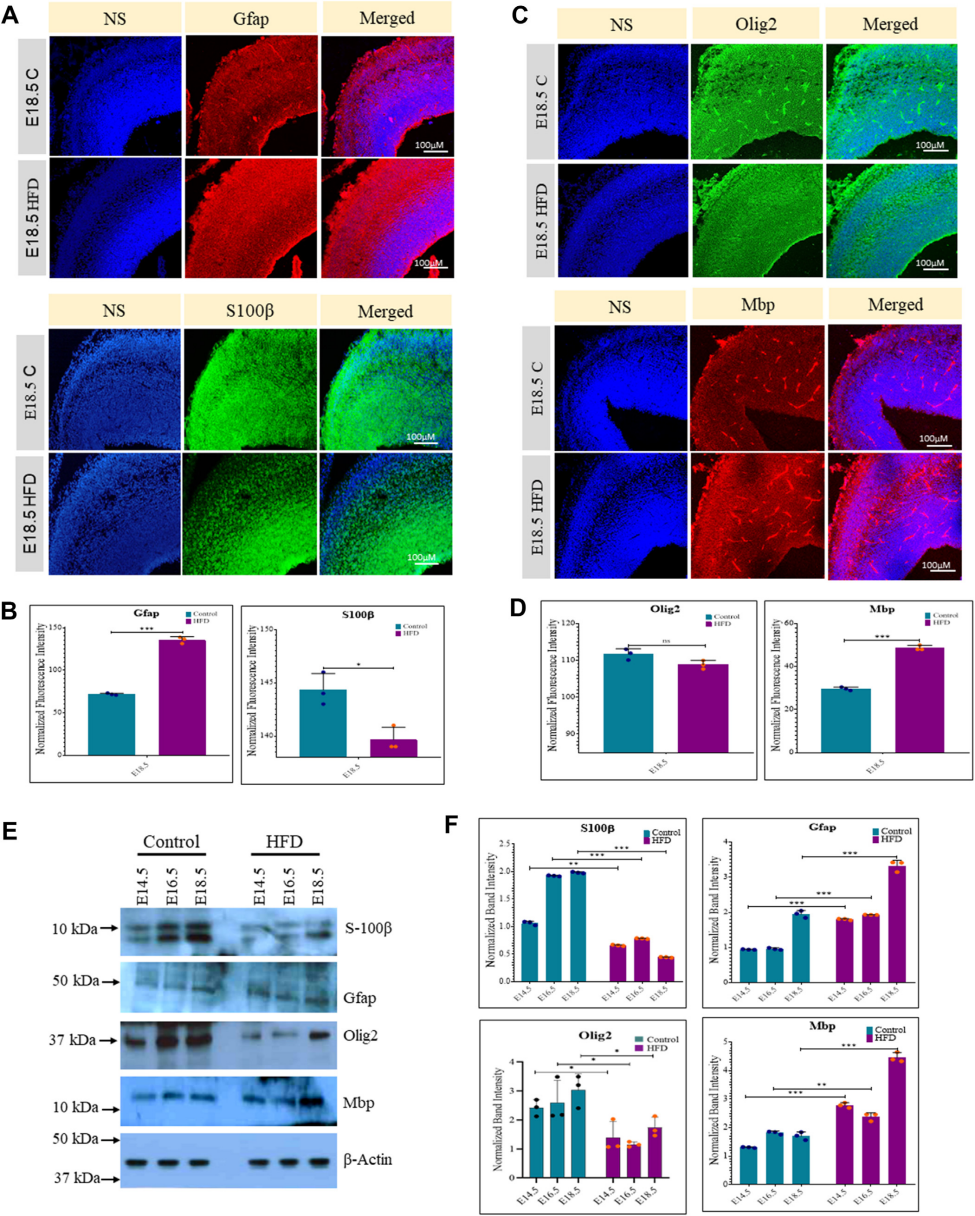
**Maternal obesity causes increased astrocyte and oligodendrocyte marker expression in the embryo brain cortex.***A*, immunostaining for the expression of astrocyte markers, Gfap and S100β in the control and HFD embryo brain cortex at E18.5 stage. The scale bar represents 100 μm. *B*, fluorescence intensity from *panel A* was quantified using ImageJ (Fiji) software and normalized to TO-PRO-3 nuclear staining intensity. *C*, immunostaining for the expression of oligodendrocyte markers, Olig2 and Mbp in the control and HFD embryo brain cortex at E18.5 stage. The scale bar represents 100 μm. *D*, fluorescence intensity from *panel C* was quantified using ImageJ (Fiji) software and normalized to TO-PRO-3 nuclear staining intensity. *E*, Western blot analysis for the expression of Gfap, S100β, Olig2, and Mbp on control and HFD embryo brain cortical tissue lysates at E14.5, E16.5, and E18.5 stages. The expression of β-Actin was used as the loading control. *F*, densitometric quantitation of Western blots from *panel E*. n = 3. The data (*bars*) are represented as mean ± SD. ∗∗*p* < 0.01 and ∗*p* < 0.05. n/s, not significant; HFD, high-fat diet.

We also analyzed the expression of astroglial markers in the control and HFD embryo cortex at E18.5 by immunostaining. Sections stained for early astrocyte marker GFAP showed increased numbers of GFAP + cells in the HFD embryo cortex compared to control, whereas pan astrocyte marker S100β showed an opposite pattern with reduced numbers of S100β+ cells in the HFD embryo cortex (Fig. 3, *A* and *B*). These changes were further confirmed through Western blot analysis of GFAP and S100β (Fig. 3, *E* and *F*). The levels of oligodendrocyte marker, OLIG2, showed minimal alterations in control *versus* HFD sections (Fig. 3, *C* and *D*). However, Western blot analysis for OLIG2 showed significantly reduced expression in the HFD cortex at all three stages of embryo development. In contrast, HFD embryo brain sections showed increased numbers of mature oligodendrocyte marker, MBP than controls, which was further confirmed through Western blot analysis, suggesting altered oligodendrocyte production in HFD embryo brain cortex compared to control (Fig. 3, *E* and *F*). In summary, these findings suggest an imbalance in the proliferation and differentiation of neural stem cells in the developing embryonic brain cortex, with increased neuronal and astroglial marker expression in embryos from rats subjected to HFD-induced obesity, compared to those on a normal diet.

### Maternal obesity disrupts developmental and AKT signaling pathways in the brain cortex of the developing embryo

**Figure 4:**
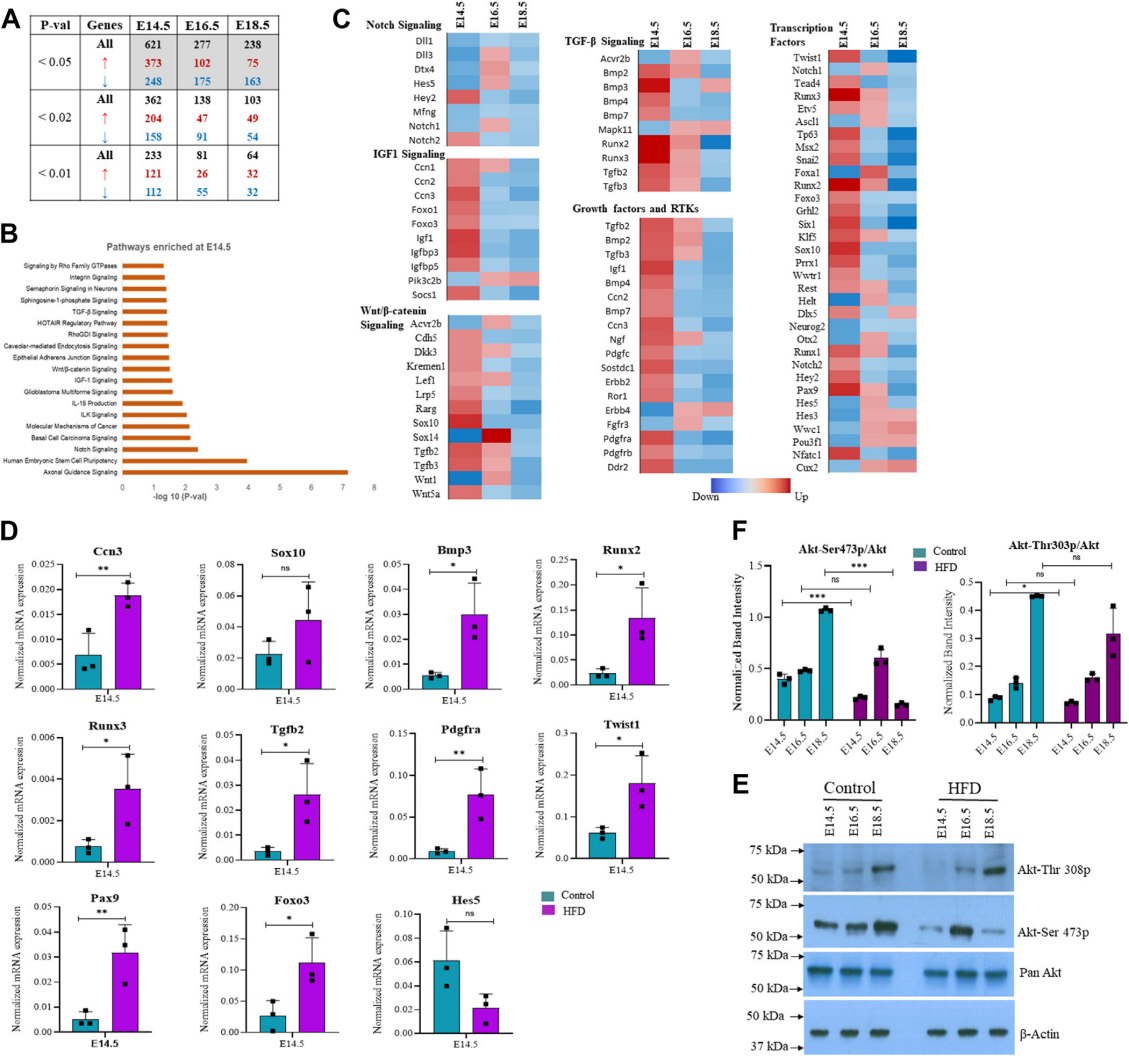
**Effect of maternal obesity on the transcriptome of embryo brain cortex.***A*, differentially expressed genes (DEGs) analysis led to the identification of genes that were upregulated or downregulated in the HFD embryo brain cortex compared to controls at E14.5, E16.5, and E18.5 stages with different *p* values. n = 2. *B*, ingenuity pathway analysis was performed on the genes significantly affected (*p* value < 0.5) in HFD embryo brain cortex compared to control at E14.5. *C*, heat map of select transcription factor and signaling pathway regulator genes identified by ingenuity pathway analysis from the list of significantly affected genes. *D*, validation of the top 11 DEGs identified from RNA-seq analysis of the E14.5 embryonic brain cortex using real-time PCR with gene-specific primers. Bar plots display the normalized Ct values of the target genes, adjusted relative to 18S rRNA, which served as the internal control. *E*, Western blot analysis for the expression of Akt-Thr308p, Akt-Ser473p, and total Akt on control and HFD embryo brain cortical tissue lysates at E14.5, E16.5, and E18.5 stages. The expression of β-Actin was used as the loading control. *F*, densitometric quantitation of Western blots from *panel D*: Akt-Thr308p and Akt-Ser473p levels are plotted relative to total Akt. n = 3. The data (*bars*) are represented as mean ± SD. ∗∗*p* < 0.01 and ∗*p* < 0.05. n/s, not significant; HFD, high-fat diet.

To understand the molecular basis of these phenotypic changes, we performed RNA-seq on the frontal lobes of embryo brains from all three developmental stages, E14.5, E16.5, and E18.5. DESeq2 was used to identify differentially expressed genes (DEGs) between control and HFD samples. The highest numbers of DEGs were identified on E14.5 with a total of 621 genes, out of which 373 genes were upregulated and 248 were downregulated significantly (*p* value 0.5). These numbers decreased subsequently at E16.5 and E18.5, showing a total of 277 and 238 DEGs, respectively (Fig. 4*A*). Pathway analysis on the list of DEGs from E14.5 showed axonal guidance signaling as the most highly enriched pathway. In addition, several major developmental signaling pathways—including the *Notch* pathway, *Wnt/β-catenin*, *Tgf-β* and *Igf1* signaling pathways—were enriched, among others (Fig. 4*B*). Interestingly, we noticed that affected genes of *Wnt/β-catenin*, *Tgf-β* and *Igf1* signaling pathways were mostly upregulated, whereas *Notch* pathway genes were mostly downregulated, specifically on E14.5. We also found that several growth factors and receptor tyrosine kinases were upregulated on E14.5. In addition, many gene-specific transcription factors (TFs) with prominent roles in embryonic neurodevelopment were also differentially expressed (Fig. 4*C*). To validate these results, we did real-time quantitative polymerase chain reaction (qPCR) analysis on 11 top DEGs, which showed changes similar to that observed in RNA-seq (Fig. 4*D*). This demonstrates that developmental signaling pathways may have been impaired in the developing embryo brain cortex of mothers on HFD.

It is well established that various growth factors and receptor tyrosine kinases signal through the AKT pathway, which plays a critical role in cell proliferation and survival.

(13, 14, 15). In our study, we found these processes to be affected in the brain cortex of embryos from HFD-exposed mothers (Figs. 1 and 2). Moreover, AKT signaling is known to regulate neurodevelopmental processes and dysregulation of its function is associated with several neurodevelopmental disorders (16, 17). Western blotting for AKT-Thr308p and AKT-Ser473p levels showed significantly reduced expression in the HFD embryo brain cortex compared to the control group at E14.5, whereas only AKT-Ser473p was reduced at E18.5. In contrast, an increase in AKT-Ser473p was observed at E16.5 in the HFD embryo brain cortex. No change was observed in the levels of total AKT (Fig. 4, *E* and *F*).

Significantly reduced AKT phosphorylation in HFD embryo brains, particularly on E14.5, reflects a feature of insulin resistance, which is often associated with obesity and an HFD (18, 19). Noticeably, these changes were dynamic and thus returned to normal or were reversed at later stages of development. These findings suggest a potential role of AKT signaling in cell proliferation/differentiation defects and cell death, which we have observed in developing embryo brains of HFD-fed rats.

### Maternal obesity alters EZH2 and histone modifications in the developing embryo brain cortex

**Figure 5:**
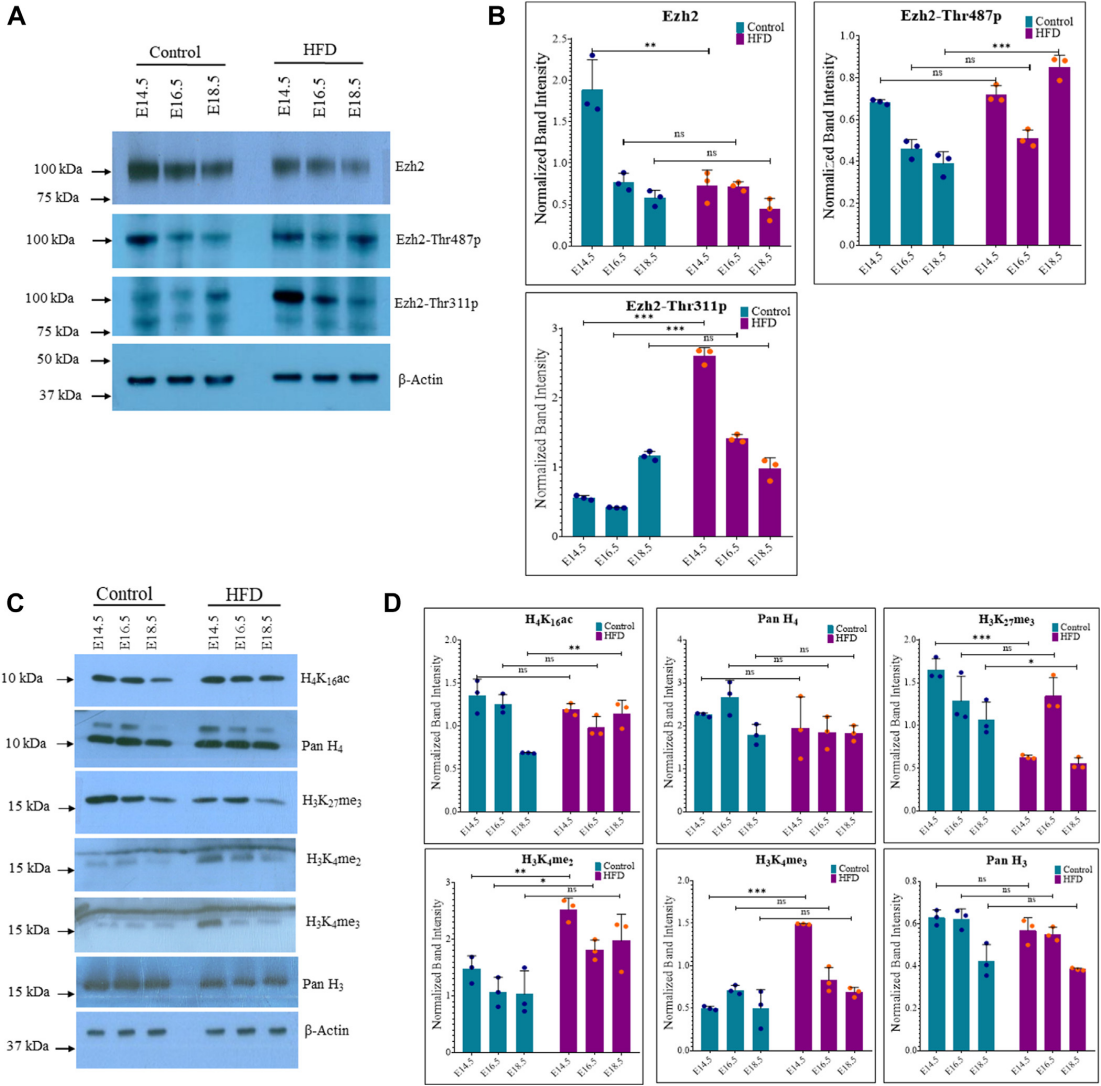
**Maternal obesity affects Ezh2 and alters global histone modifications in the embryo brain cortex.***A*, Western blot analysis for expressing Ezh2-Thr311p, Ezh2-Thr487p, and total Ezh2 on control and HFD embryo brain cortical tissue lysates at E14.5, E16.5 and E18.5 stages. The expression of β-Actin was used as the loading control. *B*, densitometric quantitation of Western blots from *panel A*. *C*, Western blot analysis for the expression of histone modifications, H3K27me3, H3K4me4, H3K4me2, pan H3, H4K16Ac, and pan H4 on control and HFD embryo brain cortical tissue lysates at E14.5, E16.5, and E18.5 stages. The expression of β-Actin was used as the loading control. *D*, densitometric quantitation of Western blots from *panel C*. n = 3. The data (*bars*) are represented as mean ± SD. ∗∗*p* < 0.01 and ∗*p* < 0.05. n/s, not significant; HFD, high-fat diet.

To identify the mechanisms of gene expression changes observed due to maternal obesity, we performed *in silico* TF enrichment analysis on the list of DEGs affected more than 2-folds. We found SUZ12 and EZH2 to be the most significantly enriched proteins for these DEGs (Fig. S1). EZH2 and SUZ12 are components of the PRC2 transcriptional repressor complex where EZH2 is the histone methyl-transferase, which transfers methyl groups to histone H3 at lysine (K)27. Trimethylation of H3 at K27 (H3K27me3) is an important epigenetic mechanism associated with gene repression and dynamically regulates key developmental genes during neurodevelopment (20, 21). Previous studies have shown that EZH2 and H3K27me3 play a role in maternal obesity–induced alterations in osteoblast and adipogenic differentiation in fetal mice (22, 23). Moreover, EZH2 is found to be phosphorylated at specific sites by various protein kinases, including AMPK and AKT (24, 25), which modulate its function. Conversely, EZH2 is found to regulate AKT phosphorylation during hippocampal neurogenesis as EZH2 deletion significantly reduced AKT phosphorylation in mice (26). Previous studies have also reported that EZH2 is essential for neural stem cell proliferation by inhibiting the expression of genes related to neuronal differentiation (27). Therefore, we checked the protein expression of EZH2 in the control and HFD embryo cortex and found significantly reduced expression at E14.5, whereas the changes were non-significant at E16.5 and E18.5 stages (Fig. 5, *A* and *B*). We then looked at two specific threonine (Thr) residues, Thr311 and Thr487 phosphorylation of EZH2, which affect its catalytic activity (24, 28) and were found to be activated due to metabolic perturbations (29). We found the levels of EZH2-Thr311p to be significantly higher in HFD embryos than controls, particularly on E14.5 and E16.5 with no significant change observed at E18.5. Whereas, EZH2-Thr487p levels showed no significant difference at E14.5 and E16.5 but were significantly upregulated at E18.5 in HFD embryos (Fig. 5, *A* and *B*).

Since EZH2 expression and its phosphorylation are altered in HFD embryos, we focused on H3K27me3 and other histone modifications known to cross-talk with it, such as H3K4me and H3K27Ac. Western blotting revealed that H3K27me3 levels were significantly reduced at E14.5 and E18.5, with no significant difference observed at E16.5. Conversely, both H3K4me2 and H3K4me3 levels were significantly elevated at E14.5. At E16.5, H3K4me2 levels remained elevated, while H3K4me3 showed no change. No differences were observed in either H3K4me2 or H3K4me3 at E18.5 in the HFD embryo brain cortex compared to controls (Fig. 5, *C* and *D*). We also examined the levels of total H4 and H4K16Ac, and although no significant changes were observed, there was a slight reduction in total H4 on E16.5 (Fig. 5, *C* and *D*). We also found levels of global H3Ac to be higher on E14.5, H3K27Ac to be higher on E14.5 and E16.5, and the levels of H3K14Ac to be higher on E14.5 in HFD embryo brain cortex. In contrast, no difference between control and HFD was observed for H3K9Ac, whereas H3K18Ac showed a subtle decrease on E18.5 (Fig. S2).

This suggested that maternal obesity causes specific histone modification alterations in the embryo brain cortex. In particular, repressive histone modification H3K27me3 is reduced on E14.5, possibly due to altered EZH2 expression and phosphorylation levels. This change was associated with H3K4me2 and H3K4me3 upregulation, a mark associated with gene activation. In addition, H3K27Ac levels were also increased, possibly also due to reduced H3K27me3. These changes raise the possibility that promoters which are kept repressed due to the H3K27me3 mark during cortical development may have been derepressed due to maternal obesity, leading to erroneous transcriptional activation observed in RNA-seq.

### Maternal obesity reduces H3K27me3 levels at promoters and transcriptional derepression in the developing embryo brain cortex

**Figure 6:**
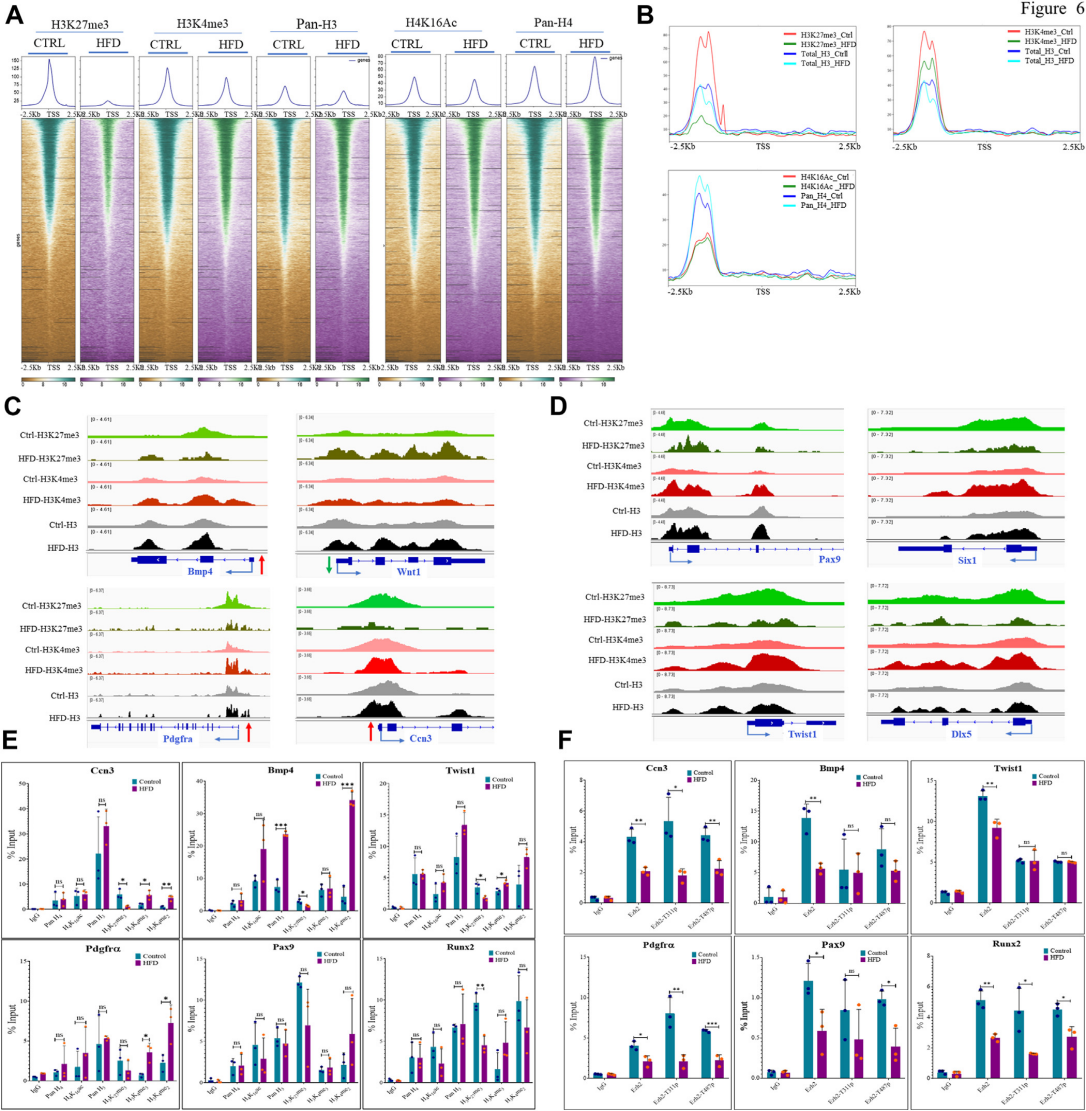
**Maternal obesity is associated with reduced H3K27me3 levels on gene promoters, contributing to transcriptional derepression.***A*, heat map of ChIp-seq data showing enrichments at −2500 bp to +2500 bp around the transcription start site (TSS) for histone H3 and H4 and modifications, H3K27me3, H3K4me3, and H4K16Ac in control and HFD embryo brain cortices from E14.5. *B*, line plots for ChIP-seq enriched peaks at −2500 bp to +2500 bp around the TSS (H3K27me3, H3K4me3, H3, and H4K16Ac, H4) on the list of genes upregulated in RNA-seq in HFD embryo brain cortices compared to control at E14.5. *C*, ChIP-seq peaks for H3K27me3, H3K4me3, pan H3 correlation with gene expression changes (*red arrow* = upregulation; *green arrow* = downregulation) observed in RNA-seq for four genes, *Bmp4*, *Wnt1*, *Pdgfra*, and *Ccn3* in control and HFD embryo brain cortices from E14.5. *D*, ChIP-seq peaks for H3K27me3, H3K4me3, pan H3 correlation with gene expression changes (upregulation) observed in RNA-seq for four noncortical TFs genes, *Pax9*, *Six1*, *Twist1*, and *Dlx5* in control and HFD embryo brain cortices from E14.5. *E*, ChIP-qPCR was done to analyze the enrichment of pan H4, H4K16Ac, pan H3, H3K27me3, and H3K4me3 at the promoter (−500 to +500 bp of TSS) of six upregulated genes, *Ccn3*, *Pgdfra*, *Bmp4*, *Twist1*, *Pax9*, and *Runx2* in control and HFD embryo brain cortices at E14.5. ChIP with IgG was used as control. Input is the total DNA. *F*, ChIP-qPCR to analyze the enrichment of the Ezh2, Ezh2-Thr311p, and Ezh2-Thr487p at the promoter (−500 to +500 bp of TSS) of six upregulated genes, *Ccn3*, *Pgdfra*, *Bmp4*, *Twist1*, *Pax9*, and *Runx2* in control and HFD embryo brain cortices from E14.5. ChIP with IgG was used as a control. Input is the total DNA. n = 3. The data (*bars*) are represented as mean ± SD. ∗∗*p* < 0.01 and ∗*p* < 0.05. n/s, not significant; HFD, high-fat diet; qPCR, quantitative polymerase chain reaction; TF, transcription factor; ChIP-seq, chromatin immunoprecipitation sequencing.

To explore the potential alteration of histone modifications in specific gene regulatory regions of chromatin due to maternal obesity, we conducted chromatin immunoprecipitation sequencing (ChIP-seq) for H3K27me3, H3K4me4, and total H3, which exhibited global changes, as well as for H4K16Ac and total H4, which remained unaltered in the HFD embryo brain cortex. ChIP was done on the embryo brain cortex (frontal lobe) dissected out on E14.5 from embryos of control and HFD rats using specific antibodies and processed for high-throughput sequencing. Peaks were called using Macs2 program. We focused on peaks enriched at −2500 bp to +2500 bp around the transcription start sites (TSS). Heat maps and line plots (for upregulated genes in HFD) showed a significantly reduced H3K27me3 levels around TSS, which correlated with global H3K27me3 changes. At the same time, H3K4me3 levels were also slightly reduced in HFD samples. Total H3 levels did not show any change. H4K16Ac levels also did not change, whereas total H4 showed a slightly increased level in HFD samples (Fig. 6, *A* and *B*). When we looked at individual genes which were found to be differentially expressed in RNA-seq, we found interesting correlations between RNA expression changes and these histone modifications. For instance, *Pdgfra*, *Ccn3*, and *Bmp4*, which exhibited upregulation in HFD samples, demonstrated decreased levels of H3K27me3 and increased levels of H3K4me3. Conversely, *Wnt1*, whose mRNA expression was decreased in HFD, displayed elevated levels of H3K27me3 in HFD samples (Fig. 6*C*). We also noticed that several developmental regulatory TFs, which normally do not express in the developing cortex during neurogenesis, were upregulated in RNA-seq; for example, *Pax9*, *Six1*, *Twist1*, and *Dlx5*. When we looked for these genes in ChIP-seq, we found H3K27me3 to be reduced with a concomitant increased enrichment of H3K4me3 (Fig. 6*D*). We also validated ChIP-seq results independently by ChIP-qPCR analysis on six upregulated genes, which showed changes similar to that observed in ChIP-seq analysis (Fig. 6*E*). In addition, we also did ChIP-qPCR analysis on the same genes for H3K27Ac modification, which we expected to be affected on target genes due to reduced H3K27me3 enrichment. Indeed, H3K27Ac was increased significantly in the HFD embryo brain cortex at the promoters of four out of six upregulated genes analyzed here compared to controls (Fig. S3).

These results suggest an imbalance of these repressive (H3K27me3) and activation (H3K4me3) histone modifications could have been directly involved in gene expression changes observed in HFD samples.

Since Western blot analysis showed reduced EZH2 levels and increased EZH2-Thr311p and EZH2-Thr487p in the HFD embryo cortex, we examined chromatin association of EZH2 and phospho-EZH2 through ChIP-qPCR on select genes which have shown upregulation in RNA-seq. Results of ChIP-qPCR analysis for the promoters of 6 genes, *Ccn1*, *Bmp4*, *Twist1*, *Pdgfra*, *Pax9*, and *Runx2*, showed reduced enrichment for EZH2 as well as both EZH2-Thr311p and EZH2-Thr487p on the HFD embryo brain cortex compared to controls (Fig. 6*F*).

Based on these findings, we infer that maternal obesity resulting from an HFD leads to transcriptional derepression, likely due to decreased levels of H3K27me3 on the promoters of target genes with a concomitant increase in active histone mark, H3K4me3 and H3K27Ac during embryonic cortical neurogenesis. These changes could have resulted from reduced chromatin association of EZH2 on target gene promoters due to reduced expression and increased phosphorylation.

These findings enable us to deduce that the chromatin dysregulations may be responsible for the identified aberrations in neurogenesis and astrogliogenesis due to maternal obesity.

### AMPK-Thr172p shows an increased association with EZH2 in the HFD embryo brain cortex

**Figure 7:**
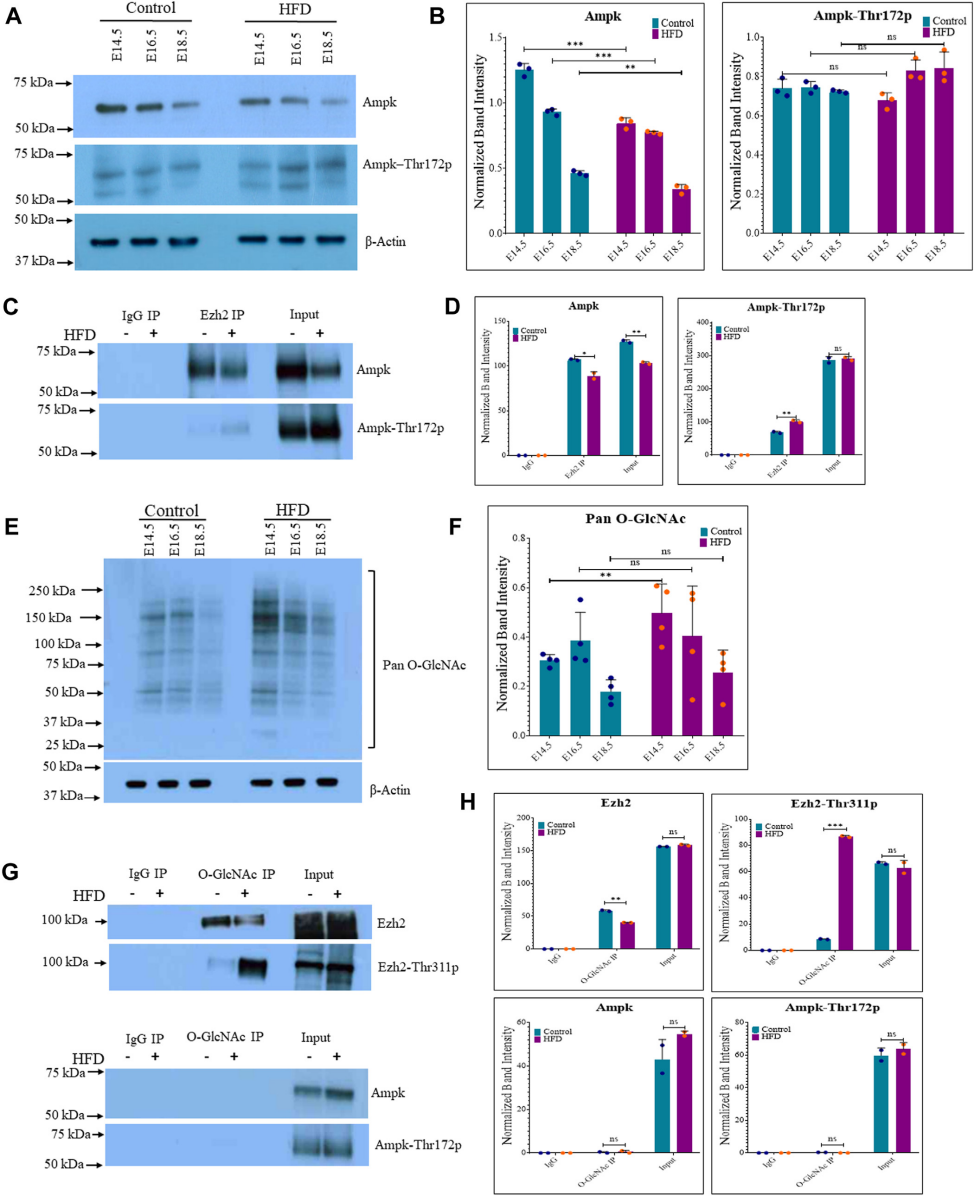
**Ampk-Thr172p and Ezh2 interaction is increased in HFD.***A*, Western blot analysis for the expression of Ampk and Ampk-Thr172p on control and HFD embryo brain cortical tissue lysates at E14.5, E16.5, and E18.5 stages. The expression of β-Actin (same as in Fig. 5*A* as the same samples were analyzed in both figures) was used as the loading control. *B*, densitometric quantitation of Western blots from *panel A*. *C*, coimmunoprecipitation was done on cell lysates from Ctrl and HFD embryo brain cortical tissue lysates from E14.5 using anti-Ezh2 antibody, followed by Western blotting of IP’d samples using anti-Ampk and Ampk-Thr172p antibodies. IP with IgG was used as a control. Input is the total cell lysate. *D*, densitometric quantitation of Western blots from *panel C*. *E*, Western blot analysis for the expression of global O-GlcNAc levels on control and HFD embryo brain cortical tissue lysates at E14.5, E16.5, and E18.5 stages. The expression of β-Actin was used as the loading control. *F*, densitometric quantitation of Western blots from *panel E*. *G*, coimmunoprecipitation was done on cell lysates from Ctrl and HFD embryo brain cortical tissue lysates from E14.5 anti-O-GlcNAc antibody, followed by Western blotting for Ezh2, Ezh2-Thr311p, Ampk, and AMPK-Thr172P. IP with IgG was used as a control. Input is the total cell lysate. *H*, densitometric quantitation of Western blots from *panels G*. Data represents the mean of three biological replicates ± SD. HFD, high-fat diet; IP, immunoprecipitation.

Previous studies on EZH2-Thr311p have identified AMPK as the kinase responsible for this particular Thr-phosphorylation of EZH2 (24). Therefore, we checked the expression of AMPK and its phosphorylated form AMPK-Thr172p in the control and HFD embryo brain cortex. A small but significant reduction in AMPK protein levels was observed in the HFD embryo brain cortex, with decreases of approximately 30% at E14.5, 17% at E16.5, and 26% at E18.5., whereas AMPK-Thr172p did not show a significant change (Fig. 7, *A* and *B*). To examine the association between AMPK and EZH2, we performed coimmunoprecipitation (Co-IP) coupled with Western blotting. We first immunoprecipitated EZH2 using an anti-EZH2 antibody from cortical tissues collected at E14.5 of control and HFD embryos, followed by Western blotting using an anti-AMPK and anti-AMPK-Thr172p antibodies. Positive bands on Western blots for AMPK and AMPK-Thr172p confirmed the association between EZH2 and AMPK and AMPK-Thr172p, whereas IgG control samples were negative (Fig. 7, *C* and *D*). HFD samples showed a reduced association between EZH2 and AMPK, possibly due to reduced levels of AMPK in HFD samples compared to controls, as observed in input bands (Fig. 7, *C* and *D*) and Western blots (Fig. 7, *A* and *B*). Interestingly, however, an increased association between AMPK-Thr172p and EZH2 was observed in HFD samples, whereas input lanes did not show a change (Fig. 7, *C* and *D*). Since AMPK-Thr172p is known to phosphorylate EZH2 at Thr311, these results suggest that increased AMPK-Thr172p and EZH2 association could have resulted in increased EZH2-Thr311p levels observed in HFD (Fig. 5, *A* and *B*). We have previously reported that maternal hyperglycemia or increasing the levels of total O-GlcNAc also increased EZH2-Thr311p levels (29). Indeed, nonfasting blood glucose levels measured in animals before sacrifice on all three stages showed mild hyperglycemia in pregnant animals on HFD, with significant differences at E14.5 and E16.5 stages (Fig. S4). Therefore, we did Western blotting to check for the global O-GlcNAc levels in control and HFD samples using an anti-O-GlcNAc antibody. We did find a significant increase (approximately 60%) in the levels of total O-GlcNAc in the HFD samples compared to control at E14.5, however no significant change was observed at E16.5 and E18.5 stages (Fig. 7, *E* and *F*). Co-IP experiments with anti-O-GlcNAc antibody coupled with Western blotting of IP’d samples for EZH2 showed a positive band on Western blot, confirming EZH2 to be O-GlcNAcylated. Interestingly, EZH2-O-GlcNAc levels were lower in HFD samples than controls. Co-IP for EZH2-Thr311p found it to be O-GlcNAcylated as well, but in contrast to EZH2, O-GlcNAc levels on EZH2-Thr311p were significantly higher in HFD samples than control, whereas, both AMPK and AMPK-Thr172p were not O-GlcNcylated (Fig. 7, *G* and *H*). These results suggest that an O-GlcNAcylated EZH2 may be needed for its increased AMPK association and its Thr311 phosphorylation in HFD, although further research is required to confirm this hypothesis.

## Discussion

Previous studies indicate that maternal HFD and obesity have an impact on brain development, resulting in both prenatal deficits and enduring effects that extend into adulthood (7, 30). However, a clear understanding of the mechanisms involved remains obscure. In this study, we used Wistar rats fed on an HFD to induce pregestational obesity in order to understand the impact of maternal HFD and obesity prior to and during pregnancy on embryonic brain development. High-fat feeding in these animals elevated plasma FFAs in pregnant rats and significantly increased lipid accumulation in the developing embryo brain cortex at the E14.5 and E18.5 stages. These changes were associated with increased cell death, as observed through TUNEL staining and cleaved PARP levels. Prior research in cell cultures and animal models has reported similar results with increased cell death in cortical neurons exposed to lipids, particularly saturated fatty acids such as palmitate (31, 32, 33). Lipid accumulation might lead to increased cell death, potentially through decreased cell survival, likely attributable to observed reductions in AKT-Thr308p and AKT-Thr472p levels. Previous studies have also observed such changes where streptozotocin-induced diabetes reduced AKT-p levels and led to apoptosis in the cerebral cortex in rats (34). AKT promotes cell survival by negatively regulating apoptosis-promoting genes such as forkhead box protein O (FOXO) TFs (35). Interestingly, we noticed that expression of both *Foxo1* and *Foxo3* were upregulated in HFD embryo brain cortex at E14.5 in RNA-seq data in our study, suggesting a mechanism through which reduced AKT-p levels could have resulted in increased cell death. In addition, AKT also promotes cell survival by directly inhibiting the regulators of the apoptosis cascade (36). Both hyperglycemia and elevated FFAs resulting from dyslipidemia are known to induce insulin resistance, which is associated with decreased AKT phosphorylation (37, 38). However, the molecular basis for insulin resistance can vary greatly due to different metabolic perturbations and their impact on target tissues. During the period of organ development, exposure to hyperglycemia or hyperlipidemia may also impact the mechanisms of cell proliferation and cell fate determination, thus affecting the process of organogenesis. Indeed, maternal obesity and diabetes are shown to impair various organ developments, including neurodevelopment (1, 39). Studies have previously reported altered hippocampal neurogenesis due to maternal obesity (40, 41, 42). Similarly, using an *in vitro* human embryonic stem cell-neuronal differentiation model, we have observed increased expression of neuronal marker β-TUBULIN III and reduced neural stem cell proliferation due to palmitate treatment (33). The present study again revealed reduced KI67 expression in the HFD embryo brain at E14.5 with a concomitant increase in neuronal differentiation marker, β-TUBULIN III. These changes were associated with altered expression of neural stem cell marker PAX6 and increased expression of differentiation regulator TBR1. Moreover, astrocyte and oligodendrocyte makers GFAP and MBP were increased with a reduction in progenitor marker, OLIG2, at the E18.5 stage in the HFD embryo cortex. Thus, our results demonstrated that maternal obesity led to increased embryo cortical neuronal production as well as early astroglial differentiation. These phenotypic changes could be attributable to changes in the protein expression and phosphorylation of EZH2, which we have observed in the HFD embryo brain cortex. Histone modifications are crucial in neurogenesis, serving as a major epigenetic mechanism for cell-type specification (43). H3K27me3 is associated with gene repression, and H3K4me3 is a mark for transcriptional activation. These are two key modifications linked to cell fate determination during development. Notably, both H3K27me3 and H3K4me3 coexist on the promoters of numerous developmental genes during neurogenesis, giving rise to the term “bivalent” promoters (44). The concept suggests that these bivalent genes, many of which are often stage-specific TFs and signaling regulators, are poised to be either activated or silenced during specific developmental stages, thereby influencing cell fate decisions. The deposition of H3K27me3 is carried out by the Polycomb group complex, catalyzed by the histone methyltransferase EZH2. Polycomb group proteins, including EZH2, play vital roles in neurogenesis by maintaining a balance between the self-renewal and differentiation of neural progenitor cells (27). Recent studies have highlighted the involvement of Ezh2 and associated histone modifications in maternal HFD and obesity on fetal organogenesis (45). For instance, maternal HFD increases H3K27me3 levels on genes like *Pthlh* and *Col2a1*, suppressing their expression and impairing chondro- and osteogenesis while simultaneously enhancing the expression of osteoblast inhibitor genes *Tnfaip3* and *Twist1* through elevated H3K27 acetylation (22). Maternal obesity was also found to enhance adipogenic differentiation in offspring during fetal development by increasing *Zfp423* expression through reduced EZH2 binding and decreased H3K27me3 levels at its promoter (23). These findings parallel the changes observed in neurogenesis in this study, where we found that the global levels of H3K27me3 were reduced significantly at the E14.5 stage, whereas H3K4me3 and H3K4me2 levels were higher in the HFD embryo brain cortex than controls. ChIP-seq analysis for H3K27me3 again showed reduced peaks in general and at specific EZH2 target genes, which have shown upregulation in RNA-seq in HFD. Whereas, H4K16Ac, another histone mark did not show a change. Interestingly, the role of EZH2 in metabolic sensing and signaling has been reported in several previous studies (24, 46). We found EZH2-Thr311p levels significantly elevated at the E14.5 stage in HFD, whereas EZH2-Thr487 was increased at E18.5. Wan *et al.*; were the first to identify EZH2 to be phosphorylated at Thr311 by AMPK in cancer cells and this phosphorylation of EZH2 suppresses its H3K27 methyltransferase activity (24). Moreover, we have recently shown that hyperglycemia and increased global protein O-GlcNAc levels increased EZH2-Thr311p in human embryonic stem cell-neuronal differentiation in cell culture leading to reduced H3K27me3 levels at several neurodevelopmental genes (29). Therefore, when we inquired whether maternal obesity increases global O-GlcNAc levels in the embryo brain cortex, we saw a subtle but significant increase in the global O-GlcNAc levels in the HFD embryo brain cortex compared to controls at E14.5. We also found EZH2 and EZH2-Thr311p to be O-GlcNcylated in embryo brain cortex and noticeably, EZH2-Thr311p O-GlcNAc levels were much higher in HFD. These results are consistent with our previous observations where increased O-GlcNAc levels were associated with elevated EZH2-Thr311p (29). AMPK serves as a widespread sensor of cellular energy and nutrient status across eukaryotic cells (47). AMPK is activated during low energy states, signaling to enhance ATP generation and reduce ATP consumption triggered by high AMP/ATP or ADP/ATP ratios and phosphorylation of AMPK-Thr172. In rodent studies, an HFD has been demonstrated to reduce the phosphorylation of AMPK at Thr172 (48, 49). Therefore, when we inquired about the relationship between AMPK and EZH2 by Co-IP, we found AMPK associates with EZH2. Although global levels of AMPK and its association with EZH2 were reduced in HFD, its association with EZH2-Thr311p increased significantly. This remarkable result lets us speculate that increased EZH2-O-GlcNAcylation could lead to its increased association with AMPK-Thr172p, thus resulting in increased EZH2-Thr311p.

**Figure 8:**
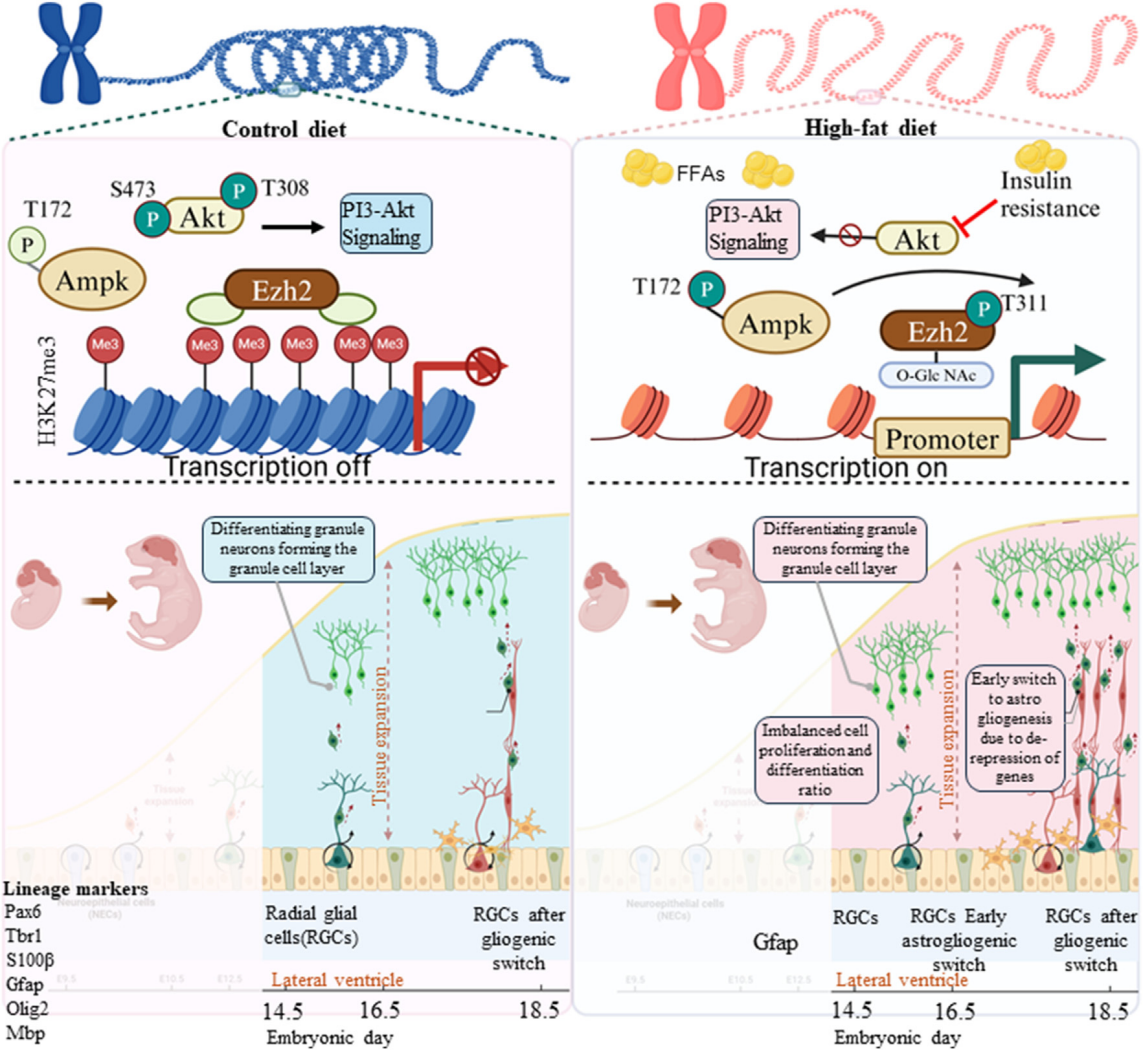
**Schematic representation of the effects of maternal obesity on the regulation of gene expression and cell fate during embryonic cortical neurogenesis**.

In conclusion, we report that maternal HFD and obesity profoundly impacted embryonic neurodevelopment resulting from an imbalance in cell proliferation and differentiation in the developing cerebral cortex. Maternal HFD led to increased cell death concomitant with increased neuronal differentiation and an increase in astrogliogenesis. These changes were associated with the differential expression of several cell signaling regulators and TFs. Moreover, reduced expression and altered phosphorylation of EZH2 and its increased O-GlcNAcylation in HFD led to reduced H3K27me3 levels and transcriptional derepression of EZH2 target genes (Fig. 8). These mechanisms might contribute to the observed adverse neurodevelopmental outcomes associated with maternal obesity in human epidemiological studies. Future research could leverage these mechanisms as a therapeutic strategy to mitigate the detrimental effects of maternal obesity–mediated metabolic perturbation in the offspring.

## Materials and methods

### Generation of the model of maternal obesity in Wistar rats and sample collection

Healthy female and male Wistar rats were obtained from the Animal House facility of the College of Medicine and Health Sciences, UAE University, and crossed to obtain a new generation of healthy and homogenous pups. The females and the male rat pups were separated into different cages after the weaning period, and the female pups were further divided into two groups—the “Control” group fed on the normal chow and the “HFD” group fed on the commercially available HFD produced by Research Diets Inc. 60% calories by fat, 20% calories by protein, and 20% calories by carbohydrate; Product No.: D14292). Rats were fed *ad libitum* with their respective feeds and water, and a 12-h dark and light cycle was maintained throughout the procedure. The weights of the female rats were monitored every week. Once the female rats of the HFD group became obese compared to the Control animals, they were crossed with healthy males. Once their pregnancy was confirmed with the presence of a plug, females of the HFD and the control groups were isolated into different cages. On days E14.5, E16.5, and E18.5 of pregnancy, different sets of control and HFD group mothers were sacrificed. The frontal lobe cortices from an entire litter of embryos (6–8) were pooled together and used as the starting material for each separate experiment, serving as an independent biological replicate for molecular studies. The tissues were snap-frozen in liquid nitrogen and stored at −80 °C until further use. Based on previous studies, embryonic stage 14.5 (E 14.5) was the stage of development at which the neurogenesis peaked, and E18.5 was the stage for the initiation of astrogliogenesis. Therefore, the tissues were decided to be harvested at these two stages and a stage in between them, E 16.5. All animal experiments were reviewed and approved by the Animal Ethics Committee of the UAE University (Approval numbers, ERA 2020-6059, ERA2022-1027). In addition, the nonfasting blood glucose level of the pregnant females was measured with a OneTouch ultraglucometer at the time of sacrifice.

### Estimation of FFAs in the control and HFD rat mother blood serum samples

With the sacrifice of each Wistar rat mother, blood samples were taken and spun immediately to separate the blood serum. The samples were briefly spun to isolate the layer of serum in the tube, which was then frozen and stored. The FFA assay kit from Abcam (ab 65,431) was used to determine the levels of FFAs in the serum samples through colorimetric detection as per manufacturer’s instructions.

### Cryo-sectioning of the Wistar rat embryo brains

During the harvesting of brain cortex tissues of the Wistar rat embryos at the embryonic stages 14.5, 16.5, and 18.5, 2 to 3 embryo brains of all three stages were saved whole by storing them in 4% paraformaldehyde for 24 h after dissection at 4 °C. The whole brains were then transferred into a 20% sucrose solution containing sodium azide to remove the water content of the brains to avoid crystal formation that may affect the sectioning. Once the brains had sunk to the bottom of the Sucrose solution in a couple of days, they were dehydrated and were ready to be embedded. Embedding molds (Polysciences, #18986, 12 mm bottom) and embedding medium (Tissue Tek - OCT compound - 34583) were used to embed the brains. The orientation of the brains during embedding was done carefully and duly labeled. The embedded sections were then stored at −80 °C until further use. Meanwhile, fresh glass slides were coated with APES or Gelatin to make them more adhesive for fixed tissue sections. The optimal cutting temperature cubes containing the frozen embryos were then sectioned using a Leica cryostat (Leica CM1800) into 5μ thick sections after removing the Olfactory bulbs. Only the prefrontal cortex was sectioned in this manner. The sections were then collected onto the coated slides, and the slides were frozen till further use.

### TUNEL staining of the cortex tissues

The embryo brain cortex sections of E14.5 Control and HFD mother rats were stained for TUNEL-positive cells using the TdT In Situ Apoptosis Detection Kit from Biotechne, R&D Systems (Cat. No.: 4810-30-CK). The slides for the positive and the negative control were prepared per the manufacturer’s guidelines, and the slides of sections were stained for comparison. The number of TUNEL-positive cells was counted in the section and expressed as a percentage of the positive control.

### Nile Red staining of the cortex tissues

Control and HFD embryo brain sections of all three embryonic stages (E14.5, E16.5, and E18.5) were subjected to Nile Red staining per the guidelines of the staining kit from Abcam (ab228553). The lipid globules uptake by the cells was stained in red in this protocol. The stained cortex tissue sections were then observed and imaged under a confocal microscope. The immunofluorescence intensity of the images of the sections was then quantified using the software Image J (Fiji; https://imagej.net/software/fiji/downloads) and plotted for comparison.

### Immunohistochemistry

The slides were incubated in 1% SDS solution for 10 min to retrieve antigens at room temperature (RT). After three wash cycles in PBS (5 min each), the endogenous peroxidase activity was blocked by placing the tissue sections in 0.4% Triton X-100 PBS, 1% bovine serum albumin (BSA), and 4% goat serum for 20 min. This was followed by incubation with specific primary mAbs (detailed in Tables 1 and 2) diluted using the same blocking solution (1:200). Following overnight incubation at 4 °C in a humidity slide moisture chamber, the slides were washed with 1X PBS and incubated with a fluorescent secondary antibody diluted (1:200) using 1% BSA and 0.3% Triton X-100 in PBS for 60 min at RT. The slides were then washed three more times with PBS. The tissues were then incubated with 4′,6-diamidino-2-phenylindole or TO-PRO3 (Blue) for 5 min at RT. After a couple more washes with 1% PBS, the slides were air-dried. Mounting media (Vectashield) was then carefully added to cover each slide section. The sections were covered by coverslips and sealed using nail polish to preserve fluorescence. All images were captured using the Nikon 80 Eclipse confocal microscope at 20X magnification. Fluorescence intensity quantification was performed using ImageJ (Fiji) software. The mean fluorescence intensity of each target marker was normalized to the corresponding 4′,6-diamidino-2-phenylindole/TO-PRO3 fluorescence intensity.

**Table 1:** List of Antibodies used for Western blotting. immunohistochemistry, ChIP, and ChIP-seq

Antibody	Source	Catalog No.	Application
OCT-4 antibody	Abcam	ab19857	Immunohistochemistry, Western blotting
Nanog (1E6C4) Mouse mAb	Cell Signaling Technology	4893	Immunohistochemistry, Western blotting
Anti-β-tubulin isotype III antibody, mouse monoclonal	Sigma-Aldrich	T-5076	Immunohistochemistry, Western blotting
Purified anti-Pax-6 antibody	Biolegend	901301	Western blotting
OTX2 (D7Y3J) rabbit mAb	Cell Signaling Technology	11943	Immunohistochemistry, Western blotting
Anti-Tbr1 antibody	Millipore	AB2261	Immunohistochemistry, Western blotting
S100B Rabbit pAb	ABclonal	A0676	Immunohistochemistry, Western blotting
GFAP Rabbit pAb	ABclonal	A0237	Immunohistochemistry, Western blotting
NKX2-2 Rabbit pAb	ABclonal	A16696	Immunohistochemistry, Western blotting
OLIG2 antibody	Proteintech	13999-1-AP	Immunohistochemistry, Western blotting
Purified anti-MBP antibody	Biolegend	836504	Immunohistochemistry, Western blotting
PARP antibody	Cell Signaling Technology	9542	Western blotting
GAPDH (14C10) Rabbit mAb	Cell Signaling Technology	2118	Western blotting
Phospho-Akt (Ser473) (D9E) XP ® Rabbit mAb	Cell Signaling Technology	4060	Western blotting
Stat3 antibody (F-2)	Santa Cruz	sc-8019	Western blotting
p-Stat3 Antibody	Santa Cruz	sc-8059	Western blotting
Anti-EZH2	Proteintech	21800-1-AP	Western blotting, ChIP
Phospho EZH2 (Thr 487)	Invitrogen	PA5-105660	Western blotting, ChIP
Phospho EZH2 (Thr 311)	Cell Signaling Technology	27888	Western blotting, ChIP
AMPK a1/AMPK a2	ABclonal	A12718	Western blotting
Phospho-AMPKα (Thr172) (40H9) Rabbit mAb	Cell Signaling Technology	2535	Western blotting
Anti-AKT1 + AKT2 + AKT3 antibody	Abcam	ab126811	Western blotting
Pan H4	Diagenode	C15410156	Western blotting, ChIP, ChIP-Seq
H4K16ac antibody	Diagenode	C15200219	Western blotting, ChIP, ChIP-Seq
Anti-Histone H3 antibody	Abcam	Ab18521	Western blotting, ChIP, ChIP-Seq
TriMethyl-Histone H3-K27	ABclonal	A2363	Western blotting, ChIP, ChIP-Seq
H3K4me2 polyclonal antibody	Invitrogen	49-1004	Western blotting, ChIP
Anti-acetyl-Histone H3 antibody	Millipore	06-599	Western blotting
Acetyl-Histone H3-K27 Rabbit pAb	ABclonal	A7253	Western blotting, ChIP
Anti-Histone H3 (acetyl K9) antibody	Abcam	Ab177177	Western blotting
Acetyl-Histone H3 (Lys14) (D4B9) Rabbit mAb	Cell Signaling Technology	7627	Western blotting
Anti-Histone H3 (acetyl K18)	Abcam	Ab1191	Western blotting
Peroxidase AffiniPure Goat Anti-Mouse IgG, Fcγ Subclass 2b-specific	Jackson Immuno Research	115-035-207	Western blotting
Peroxidase AffiniPure Goat Anti-Mouse IgG (H + L)	Jackson Immuno Research	115-035-166	Western blotting
Peroxidase AffiniPure Goat Anti-Rabbit IgG (H + L)	Jackson Immuno Research	111-035-144	Western blotting
Peroxidase AffiniPure Rabbit Anti-Chicken IgY (IgG) (H + L)	Jackson Immuno Research	303-035-003	Western blotting
Anti-mouse IGg2b gamma chain (Alexa Fluor 488)-Rat monoclonal	Abcam	ab172326	Immunohistochemistry
Goat Anti-chicken IgY H&L (Alexa Fluor 488)	Abcam	ab150173	Immunohistochemistry
Goat Anti-Rabbit IgG H&L (Alexa Fluor® 594) preadsorbed	Abcam	ab150080	Immunohistochemistry
Goat Anti-Mouse IgG H&L (Alexa Fluor® 594) preadsorbed	Abcam	ab150120	Immunohistochemistry
Goat Anti-Mouse IgG H&L (Alexa Fluor® 488) preadsorbed	Abcam	ab150117	Immunohistochemistry
Goat Anti-Rabbit IgG H&L (Alexa Fluor® 488) preadsorbed	Abcam	ab150081	Immunohistochemistry

ChIP, chromatin immunoprecipitation; ChIP-seq, chromatin immunoprecipitation sequencing; OCT, optimal cutting temperature.

**Table 2:** List of Primers used for real time qPCR

Primer name	5′-3′ sequence
Igf1-Forward	5′-CCCGGGACGTACCAAAATGA-3′ (20mer)
Igf1-Reverse	5′-GCTGGTAAAGGTGAGCAAGC-3′ (20mer)
Igfbp3-Forward	5′-GGACCTTCCTTTGTCAGGCA-3′ (20mer)
Igfbp3-Reverse	5′-TAAGCGCACCGTTATTTGCG-3′ (20mer)
Ccn3-Forward	5′-AGCCTACAGACCGGAAGCTA-3′ (20mer)
Ccn3-Reverse	5′-TCCACAGCTCTTGGAACACG-3′ (20mer)
Bmp3-Forward	5′-AATCACGCCACCATCCAGAG-3′ (20mer)
Bmp3-Reverse	5′-CAAGATGCTGAGCGAGGACA-3′ (20mer)
Runx2-Forward	5′-CAAGGAGGCCCTGGTGTTTA-3′ (20mer)
Runx2-Reverse	5′-AGAACTTGTGCCCTCTGTTGT-3′ (21mer)
Runx3-Forward	5′-CACTGGCGCTGCAACAAG-3′ (18mer)
Runx3-Reverse	5′-CGGCGGAGTAATTCTCGTCA-3′ (20mer)
Tgfb2-Forward	5′-CGAAACTGTCTGCCCAGTTG-3′ (20mer)
Tgfb2-Reverse	5′-TGTAGAAAGTGGGCGGGATG-3′ (20mer)
Twist1-Forward	5′-TCGGACAAGCTGAGCAAGATT-3′ (21mer)
Twist1-Reverse	5′-GCAGCTTGCCATCTTGGAGT-3′ (20mer)
Foxo3-Forward	5′-AGACGACGAGGACAGCGG-3′ (18mer)
Foxo3-Reverse	5′-GGCCGAATCTTCGAGGAGC-3′ (19mer)
Pax9-Forward	5′-TGTTCCCGTGTGGTGAGAAG-3′ (20mer)
Pax9-Reverse	5′-CAAGGCTCTGTACTGCGTGA-3′ (20mer)
Igf1-Forward	5′-TCGTCTAGGCCATTAGCCCT-3′ (20mer)
Igf1-Reverse	5′-GGCGTTAAAACTTGGGTGGG-3′ (20mer)
Foxo3-Forward	5′-GGAACGTGATGCTTCGCAAC-3′ (20mer)
Foxo3-Reverse	5′-GGTGGTGGAGCAAGTTCTGA-3′ (20mer)
Rarg-Forward	5′-TCCCCTCCAGCAGTTTCTACC-3′ (21mer)
Rarg-Reverse	5′-TCCAATGGGTCTCCAAGGGTC-3′ (21mer)
Sox10-Forward	5′-CGATGACAAGTTCCCGGTGT-3′ (20mer)
Sox10-Reverse	5′-TCTTGCTGGCACCGTTGA-3′ (18mer)
18SrRNA-Forward	5′-AGTCCCTGCCCTTTGTACACA-3′ (21mer)
18SrRNA-Reverse	5′-GATCCGAGGGCCTCACTAAAC-3′ (21mer)

qPCR, quantitative polymerase chain reaction.

### Protein extraction and quantitation

Rat embryo brain cortex tissues were thawed, cut into smaller pieces, and weighed (40–50 mg). The rat cortex tissues were homogenized in 1X radioimmunoprecipitation assay buffer buffer containing 1X HALT Protease and 1X HALT phosphatase inhibitor Cocktail (Thermo Fisher Scientific, Cat. #1862209, Cat. #1862495). The crude brain tissue lysate was centrifuged at 8000 rpm at 4 °C for 30 min. The supernatant containing total cellular protein was collected, and the concentration was determined using the Pierce BCA Protein Assay Kit (Cat. #23225). Quantitation was performed using the Infinite M200Pro (TECAN) multimode microplate reader. The quantitated protein was denatured by heating it in a NuPAGE lithium dodecyl sulfate sample buffer (Life Technologies, NP0007) for 10 min at 70 °C. Forty micrograms aliquots of the protein in the sample buffer were frozen at −80 °C until use.

### SDS-PAGE and Western blotting

Protein loading concentrations were determined based on the analyzed target (typically 20–40 μg). Precision Plus Protein Dual Color Standards (Bio-Rad, Cat. #1610374) were loaded on Express Plus 4 to 12% PAGE gels (GenScript, Cat. #M41210 or M41215). The electrophoresis was carried out in 1X MOPS buffer (GenScript, M00138) at 100 V, after which electroblotting was performed in 1X Towbin's buffer at a constant voltage of 100 V for 1 h at 4 °C. Polyvinylidene fluoride membranes (Thermo Fisher Scientific, Cat. # 88518), to which the proteins were transferred, were blocked with 5% Blotto milk (Santa Cruz Biotechnology, sc-2324) for 1 h at RT. Alternatively, 5% BSA is used to block the membranes specifically targeted for phosphorylated proteins. Membranes were washed several times in 1X PBS, containing 0.05% Tween 20 and were incubated overnight at 4 °C with primary antibodies (details provided in Tables 1 and 2). Membranes were probed with peroxidase-conjugated secondary antibodies incubating them for 1 h at RT. Chemiluminescent substrates (West Plus Pico and West Plus Femto from Thermo Fisher Scientific, Cat. #34080 and 34096)) were used for band visualization. Blots were exposed to autoradiography films (Santa Cruz, Cat. # sc-201697) and developed using Carestream GBX Developer (Cat. #1900984) and Fixer (Cat. #1902485) solutions purchased from Sigma. The Western blot analysis revealed specific protein bands corresponding to the targeted proteins in rat embryo brain cortex tissues. Band intensities were quantified using Image J software.

### RNA isolation and RT-qPCR

Total RNA was extracted following the guidelines stipulated in the MasterPure RNA Purification Kit (Epicentre, Cat. #MCR 85102). The complementary DNA (cDNA) synthesis was executed utilizing 1.0 μg of total RNA in a 20 μl reaction. Based on the manufacturer's prescribed protocol, the SuperScript VILO cDNA Synthesis Kit (Cat. #11754050) was employed. Real-time qRT-PCR was systematically conducted, employing SYBR Green Real-Time PCR Master Mix (Thermo Fisher Scientific) in a 10 or 20 μl reaction volume. The primer sequences utilized for qRT-PCR are listed in Table 2.

### RNA-seq and data analysis

Total RNA from E14.5, E16.5, and E18.5 rat embryo cortex tissues was isolated using the MasterPure RNA Purification Kit from Epicentre (Cat. #MCR 85102) following the manufacturer's guidelines. Subsequently, the RNA libraries for sequencing were prepared using the NEBNext Ultra II RNA Library Prep Kit for Illumina (NEB #E7775) as per the kit's instructions. During the library preparation process, 1 μg of total RNA was utilized to eliminate ribosomal RNAs with the NEBNext rRNA Depletion Kit v2 (Human/Mouse/Rat) (E7400L), and the resulting RNA samples were purified using AMPure XP beads from Beckman Coulter. The purified RNA samples were then subjected to RNA fragmentation, cDNA synthesis, adapter ligation, and purification, following the kit instructions, to construct the libraries. The adapter was then ligated to the cDNA, and the adapter-ligated cDNA was subjected to PCR amplification using NEBNext Multiplex Oligos for Illumina (E6440S), incorporating specific combinations of i5 and i7 index primers for multiplexing. A final purification step using AMPure XP beads was carried out for the libraries, and they were subsequently assessed for quality before sequencing. Sequencing was performed at the Genomics Core Facility, The Wistar Institute on Illumina Next-Generation Sequencer (NextSeq 500), followed by bioinformatic analysis. RNA-seq data were aligned using bowtie2 (https://bowtie-bio.sourceforge.net/bowtie2/index.shtml) (50) algorithm against RGSC Genome Assembly v6.0, and RSEM v1.2.12 (https://github.com/deweylab/RSEM) software (51) was used to estimate read counts and fragments per kilobase of transcript per million fragments mapped values using gene information from Ensemble transcriptome version GRCh37.p13. Raw counts were used to estimate the significance of differential expression differences between experimental groups using DESeq2 (https://bioconductor.org/packages/release/bioc/html/DESeq2.html) (52). Overall gene expression changes were considered significant if passed false discovery rate (FDR) <5% unless stated otherwise. Gene set enrichment analysis was carried out using QIAGEN’s Ingenuity Pathway Analysis software (QIAGEN Redwood City, www.qiagen.com/ingenuity) using “canonical pathways” and “upstream regulators” options. Expression heat maps were generated using DESeq2 normalized count values. The significance of the overlap between stages was tested using Fisher’s exact test. DEGs with a 2-fold or more change with a *p* value of ≤0.5 were considered significant. Hierarchical clustering was performed using Euclidean distance to visualize the expression of genes across the control group and the HFD group samples that were significant in at least one sample type with FDR < 5%.

### ChIP and ChIP-qPCR

The harvested embryo brain cortex tissues from different time points were thawed as required. About 50 to 80 mg of dissected embryo brain cortices obtained from specific stages of pregnancy were subsequently washed, followed by lysis in a cell lysis buffer. The complete lysis of the tissues was achieved using fresh syringes. The chromatin was fragmented *via* sonication using a Diagenode bioruptor for 12 min, employing 30-s on/off cycles. Immunoprecipitation of the lysate was carried out using ChIP-grade antibodies, with details on the antibodies utilized provided in Table 1. The protein–chromatin complexes were collected using protein A or B Magna ChIP magnetic beads (Millipore). ChIP with normal IgG (Millipore, Cat # PP64B) was employed as a negative control. Subsequently, ChIP'd DNA was recovered and subjected to analysis *via* RT-qPCR utilizing SYBR Green Real-Time PCR Master Mix (Thermo Fisher Scientific) within a 10 μl reaction volume. The primer sequences employed for amplifying promoter or coding regions can be found in Table 3.

**Table 3:** List of Primers used for ChIP-qPCR

Primer name	5′-3′ sequence
Igf1-Ch-Forward1	5′-ATAAACTCGCTCCCGTGTCC-3′ (20mer)
Igf1-Ch-Reverse1	5′-TTAACTTTCCGCAGGGCTCG-3′ (20mer)
Ccn3 -Ch-Forward1	5′-ATGAGCGTCTTCCTGCGAAA-3′ (20mer)
Ccn3 -Ch-Reverse1	5′-ATCCCGGGCATTTTAAGCGT-3′ (20mer)
Sox10-Ch -Forward1	5′-AAAGGGGCAGCGATGTGTTA-3′ (20mer)
Sox10-Ch -Reverse1	5′-ACCCTTTGCTCCAGCGATAC-3′ (20mer)
Bmp3-Ch-Forward2	5′-CTCCTCGGTTCTCATCGCAC-3′ (20mer)
Bmp3-Ch-Reverse2	5′-AGGTAGACGAGTTTGGGCTG-3′ (20mer)
Runx2-Ch-Forward1	5′-GCGGTGCAAACTTTCTCCAG-3′ (20mer)
Runx2-Ch-Reverse1	5′-GAGCACTCACTGACTCGGTT-3′ (20mer)
Tgfb2-Ch-Forward2	5′-TGGCTGGCTCACTAACTATCC-3′ (21mer)
Tgfb2-Ch-Reverse2	5′-GCCATGATTTGGCTCTCTGGT-3′ (21mer)
Twist1-Ch-Forward1	5′-ACTTCGAAAAGTCCCTCCTCC-3′ (21mer)
Twist1-Ch-Reverse1	5′-CTTGGCGGTTCTTATACCTGC-3′ (21mer)
Foxo3-Ch-Forward1	5′-CAGTCCACACCTTTTGGTGC-3′ (20mer)
Foxo3-Ch-Reverse1	5′-AACACCCGAGATAAGACGCC-3′ (20mer)
Pax9-Ch-Forward1	5′-GTTGTATGCACACGCCAAGG-3′ (20mer)
Pax9-Ch-Reverse1	5′-CACGGCCCTCGTCTGTTAAA-3′ (20mer)
Pdgfra-Ch-Forward1	5′-CAAATGTGGGGCACACGAAT-3′ (20mer)
Pdgfra-Ch-Reverse1	5′-AGAACGTTCAGTGTTTGCCG-3′ (20mer)
Igfbp3-Ch-Forward1	5′-ATGTCCCATCGATGCCAAGG-3′ (20mer)
Igfbp3-Ch-Reverse1	5′-GTCTGGACATAGGAAACCATGA-3′ (22mer)
Rarg-Ch-Forward1	5′-AAAGTCCATTAGCAGCTCCACC-3′ (22mer)
Rarg-Ch-Reverse1	5′-GAAACTCAACAGGGCGTGGAT-3′ (21mer)
BmpP4-Ch-Forward1	5′-GTAAGAATGGAGAGCAGTCGCC-3′ (22mer)
Bmp4-Ch-Reverse1	5′-GGGCTGGGGGTAGACTGA-3′ (18mer)
DIx5-Ch-Forward1	5′-GCAAGTGCCTGTGTGAGATG-3′ (20mer)
DIx5-Ch-Reverse1	5′-GGGCCCGAAGTTGGGTAGTA-3′ (20mer)
FoxA1-Ch-Forward1	5′-GACACAGAAGGCTCACCCC-3′ (19mer)
FoxA1-Ch-Reverse1	5′-AGCAGGTGGGTAATCTGACA-3′ (20mer)

ChIP, chromatin immunoprecipitation; qPCR, quantitative polymerase chain reaction.

Primers highly specific to the promoter regions of the differentially expressed genes were designed and used for real-time qPCR analysis of the two types of E14.5 cortex tissue samples whose related chromatin regions were pulled down by the histone markers, such as pan H4, H4k16ac, H3K27me3, pan H3, and H3K4me3. In addition, H3K4me2, H3K4ac, EZH2, and its phosphorylated forms at Threonine 311 and 487 were also used for chromatin precipitation based on their suspected role in the regulation of transcription due to maternal obesity.

### Coimmunoprecipitation

Co-IP experiments were conducted following established methods (29). E14.5 embryo cortex tissues were washed with 1X PBS and lysed using freshly prepared Co-IP lysis buffer. The lysates were incubated at 4 °C on a rotating platform for 30 min and then centrifuged at 12,000 rpm for 15 min. The resulting supernatant was transferred to a new tube and kept on ice. Protein concentration was determined using the Thermo Scientific BCA protein assay kit (Cat. #23225). For each immunoprecipitation reaction, 700 μg of protein lysate was combined with 5 μg of the desired antibody and incubated overnight at 4 °C on a rotating platform. After the overnight incubation, 40 μl of precleared protein A agarose beads (Millipore #16-125) were added to a total volume of 500 μl of lysate. As a negative control, a normal rabbit IgG antibody (Bio-Rad Purified Rabbit IgG Cat. #PRABP01) was included in a volume of 500 μl and incubated for 2 h at 4 °C. Additionally, 40 μg of total protein was used as input. Following incubation, the bead/antibody-protein lysate complex underwent four washes with Co-IP buffer. In each wash, beads were gently mixed on a rotating platform for 3 min, and the antibody/bead/lysate complex was separated using a microspin. The supernatant was carefully discarded without disturbing the agarose beads. The beads were resuspended in 40 μl of 2X loading dye sample buffer, denatured at 95 °C for 5 min, and then spun down after elution to separate the protein complex from the agarose bead. After the spin, the protein complex in the supernatant was transferred to a new tube and stored at −80 °C for subsequent protein expression analyses. Blot quantification was performed using ImageJ software.

### ChIP-seq and data analysis

The 2-day ChIP protocol was carried out efficiently using the relevant antibodies for the required pulldowns as described above. ChIP-seq libraries were prepared using NEBNext Ultra II for DNA Library Prep (NEB #E7645) as per the manufacturer’s instructions. Briefly, ChIP’d DNA was mixed with NEBNext Ultra II End Prep Enzyme Mix and reaction buffer to prepare the DNA ends for further processing. A suitable dilution of the NEB adaptor was then chosen depending on the initial concentration of the samples and added to the end-prepped DNA mixture. The mixture was processed in a thermocycler for ligation reaction, followed by a cleanup process with AMPure XP beads to remove unwanted material. A second round of bead-based purification ensures high-quality DNA. PCR amplification was then performed following the manufacturer’s instructions using NEBNext Multiplex Oligos for Illumina (E6440S), incorporating specific combinations of i5 and i7 index primers for multiplexing. The amplification parameters were carefully controlled to minimize unwanted by-products. A final cleanup step was carried out using AMPure XP beads to ensure the library's quality. Additionally, two more rounds of bead-based purification were performed to refine the DNA library. The DNA was then eluted from the beads and subjected to quality control using a bioanalyzer before proceeding with the next-generation sequencing. The ChIP-seq data was processed for quality check and aligned against the rat genome (rn6) using bowtie2. Aligned data was processed to identify histone modification enrichments using recommended options for macs2 and deepTools (53, 54). The targeted histone modifications and transcription factor binding were studied on the TSSs of the differentially expressed genes identified in RNA-seq. The enriched regions (peaks) were identified, and comparisons were made between the cortex tissues of rat embryos from obese mothers and control mothers. Heat maps and profile plots for enriched regions were visualised using plotHeatmap and profile Ple. Integrated Genome Browser (UCSC_IGV) was used for the features and enriched tracks visualization.

### Statistical analysis

All the experiments were performed in triplicates. Results shown are mean ± SD. Statistical analyses were made using GraphPad prism 8 software (https://www.graphpad.com/scientific-software/prism/) with asterisks representing differences being significant (∗*p* ≤ 0.05, ∗∗*p* ≤ 0.01, and ∗∗∗*p* ≤ 0.001). A one-way ANOVA was performed to assess the differences between the groups, along with Benjamini and Hochberg FDR correction, however, pairwise comparisons were performed specifically between the “Control” and the “HFD-” treated groups within the three embryonic stages E 14.5, E16.5, and E18.5. Even though we were interested in any age-dependent variations during development among the embryos, our primary focus was to analyze the influence of the altered maternal diet on the embryo cortex development, irrespective of the age of the embryos. It is for this reason we have highlighted a pairwise comparison between the “Control” and “HFD” groups within the embryonic stages in our results. It should also be noted that in the initial stages of our research, we investigated data from all three embryonic stages. However, after establishing that many of the variations due to maternal HFD were observed at the E14.5 stage embryos, we decided to focus more on this early stage.

## Data availability

Raw and processed RNA-seq data were deposited to the NCBI GEO database under accession number GSE256004, and ChIP-seq data was deposited to the NCBI GEO database under accession number GSE255917.

## Supplemental information

Supplementary information includes four supplementary figures with figure legend and three supplementary tables.

## Conflict of interest

The authors declare that they have no conflicts of interest with the contents of this article.
