# Supplementary material for: Maternal obesity alters histone modifications mediated by the interaction between EZH2 and AMPK, impairing neural differentiation in the developing embryonic brain cortex
Source: J Biol Chem. 2025 Jan 10;301(2):108173. doi: 10.1016/j.jbc.2025.108173 (PMC11847741; doi:10.1016/j.jbc.2025.108173)
Supplement: Supplementary information [file mmc1.docx]

Figure S1. Transcription factor (TF) enrichment analysis identifies PCR2 complex components, SUZ12 and EZH2 enriched at genes upregulated in embryo brain cortex due maternal HFD. A web based transcription factor enrichment analysis (TFEA) tool (https://www.iib.uam.es/TFEA.ChIP/) was used to identify TF enrichment on the list of genes significantly upregulated (≥ 2 folds with p≤ 0.5) in RNA-seq from E14.5 embryo brain cortex.


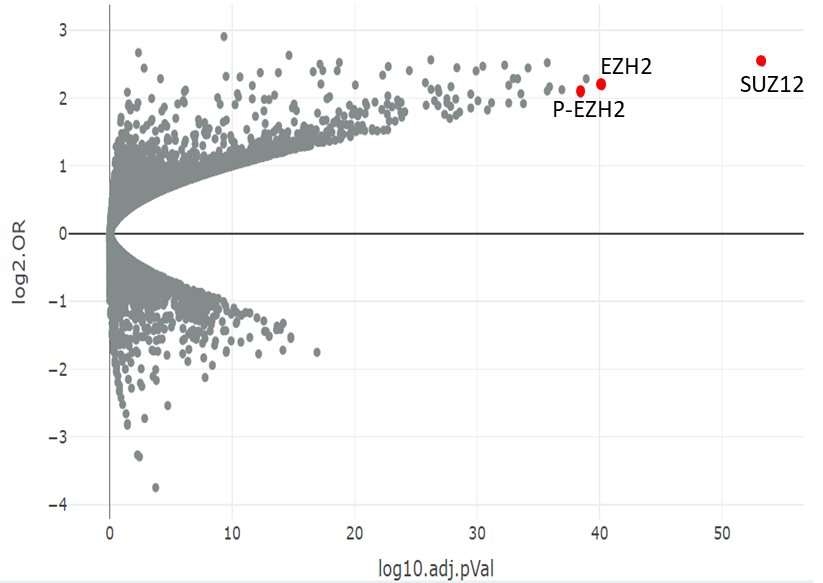


Figure S2. The effects of maternal obesity on global histone modifications in embryo brain cortex. Western blot analysis for the expression of histone modifications, H3K27Ac, H3K9Ac, H3K14Ac, H3K18Ac, pan H3Ac and pan H3 on control and HFD embryo brain cortical tissue lysates at E14.5, E16.5 and E18.5 stages. The expression of β-Actin was used as loading control (left panel). Densitometric quantitation of western blots (right panel). n=3. The data (bars) are represented as mean ± standard deviation. ** p < 0.01, * p < 0.05, n/s - not significant.


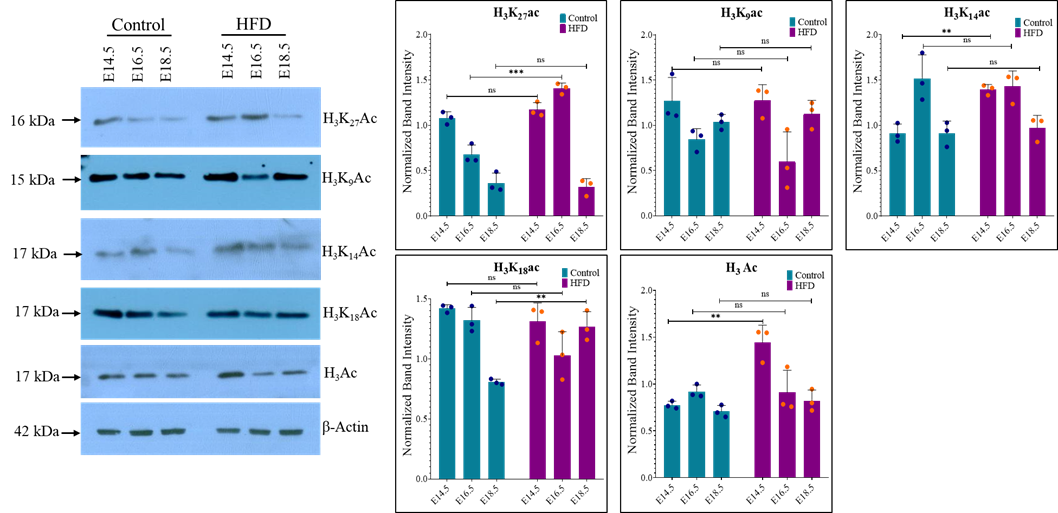


Figure S3. ChIP enrichment for H3K27Ac on select genes targets. ChIP-qPCR to analyze the enrichment of H3K27Ac at the promoter (−500 to +500 bp of TSS) of six upregulated genes, Ccn3, Pgdfra, Bmp4, Twist1, Pax9 and Runx2 in control and HFD embryo brain cortices from E14.5. ChIP with IgG was used as control. Input is the total DNA. n=3. The data (bars) are represented as mean ± standard deviation. ** p < 0.01, * p < 0.05, n/s - not significant.


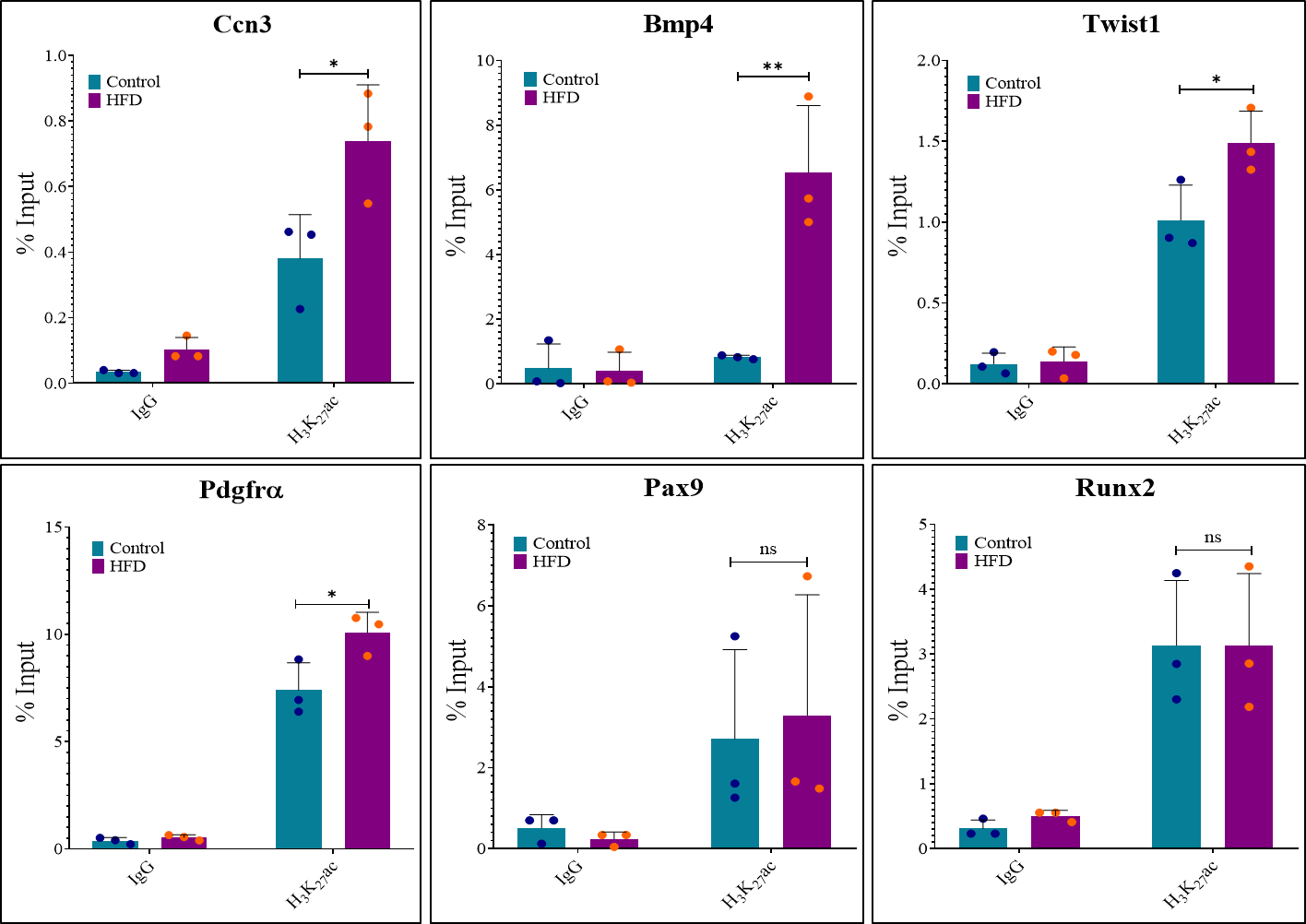


Figure S4. Random Blood Glucose Levels of control and high fat diet fed pregnant (embryonic stages 14.5, 16.5 and 18.5) Wistar rats (at the time of sacrifice). The data (bars) are represented as mean ± standard deviation. ** p < 0.01, * p < 0.05, n/s - not significant.
